# Diels–Alder Cycloaddition to the Bay Region of Perylene and Its Derivatives as an Attractive Strategy for PAH Core Expansion: Theoretical and Practical Aspects

**DOI:** 10.3390/molecules25225373

**Published:** 2020-11-17

**Authors:** Aneta Kurpanik, Marek Matussek, Piotr Lodowski, Grażyna Szafraniec-Gorol, Michał Krompiec, Stanisław Krompiec

**Affiliations:** 1 Institute of Chemistry, Faculty of Science and Technology, University of Silesia, Bankowa 14, 40-007 Katowice, Poland; aneta.kurpanik@gmail.com (A.K.); matussekmarek@gmail.com (M.M.); piotr.lodowski@us.edu.pl (P.L.); grazyna.szafraniec-gorol@us.edu.pl (G.S.-G.); 2 Merck Chemicals Ltd., 1 Venture Road, Southampton SO16 7NP, UK; michal.krompiec@gmail.com; 3 School of Chemistry, University of Southampton, Highfield, Southampton SO17 1BJ, UK

**Keywords:** APEX, perylene, bay region, Diels–Alder cycloaddition, nanographenes

## Abstract

PAHs (polycyclic aromatics hydrocarbons), the compound group that contains perylene and its derivatives, including functionalized ones, have attracted a great deal of interest in many fields of science and modern technology. This review presents all of the research devoted to modifications of PAHs that are realized via the Diels–Alder (DA) cycloaddition of various dienophiles to the bay regions of PAHs, leading to the π-extension of the starting molecule. This type of annulative π-extension (APEX) strategy has emerged as a powerful and efficient synthetic method for the construction of polycyclic aromatic hydrocarbons and their functionalized derivatives, nanographenes, and π-extended fused heteroarenes. Then, [4 + 2] cycloadditions of ethylenic dienophiles, -N=N-, i.e., diazo-dienophiles and acetylenic dienophiles, are presented. This subject is discussed from the organic synthesis point of view but supported by theoretical calculations. The possible applications of DA cycloaddition to PAH bay regions in various science and technology areas, and the prospects for the development of this synthetic method, are also discussed.

## 1. Introduction

**Figure 1:**
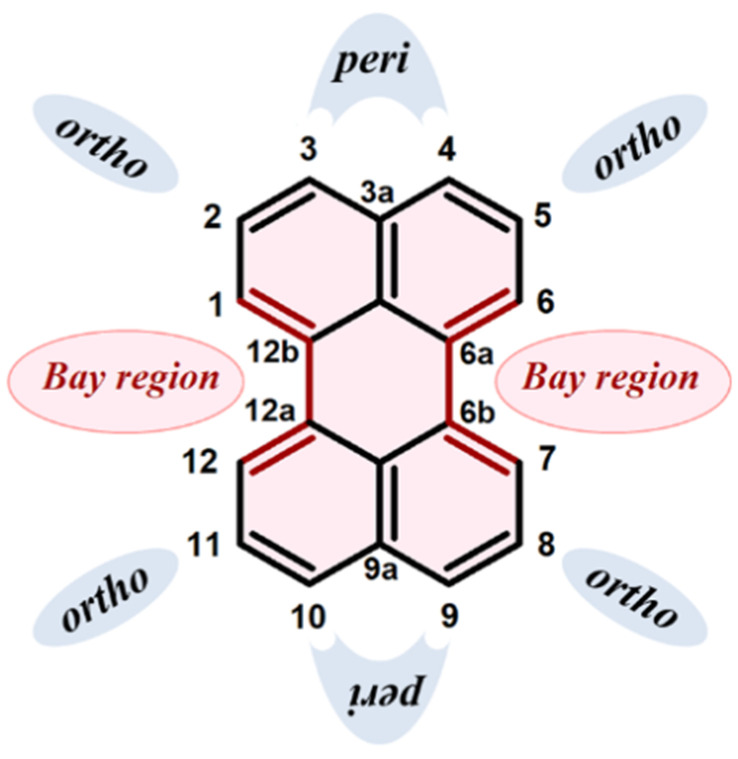
Structure and specific positions of perylene.

A great deal of research interest has been shown in PAHs (polycyclic aromatic hydrocarbons), which include compounds such as perylene, benzoperylene, coronenes, ovalenes, naphthoperylene, bisanthene, and other hydrocarbons based on the perylene structure, and various functionalized derivatives of the aforementioned molecules. Compounds of this type are of particular interest in such areas as chemistry and material science and in modern technology [1,2,3,4,5,6,7,8,9,10,11,12,13,14,15,16,17,18,19,20]. It is worth mentioning that perylene itself has persisted as an important unit in organic material chemistry, especially in dye chemistry, for over a century [21,22,23,24,25]. With respect to infinite graphene, PAHs show non-zero tunable bandgaps, and for this reason, they are applied in antenna chromophores [26,27,28] or emissive molecular architectures [29,30,31,32] and generally in all optoelectronic applications, which require a tunable semiconducting material [4,33]. PAHs, not only pure hydrocarbon but also heteroatom-doped π-scaffolds, are precursors of extended carbon networks, and their carbon (or carbon–heteroatom) skeletons can be considered small pieces of graphene [4,34,35,36,37]. In view of this, PAHs have been used in numerous theoretical and experimental studies as models for small-size graphene (i.e., nanographene) [38,39]. As has been pointed out, PAHs, particularly perylene bisimide derivatives, are of ever-increasing importance in the design and synthesis of materials for optoelectronic devices, such as solar cells [40,41,42], light-emitting diodes [43,44], field-effect transistors (FETs) [45,46], and molecular electronics [34]. Perylene derivatives are also essential components of hybrid inorganic/organic material systems, which are often more attractive than “pure” inorganic or organic materials [47,48]. Perylene imides (PIs) and bisimides (PBIs) are the most frequently researched because of their attractive properties; i.e., they are outstandingly multifaceted organic chromophores, which makes them extremely useful materials in the field of organic electronics [16,18,49,50,51,52,53,54,55,56,57,58,59,60,61,62], organic photovoltaics [63,64,65,66,67,68], and photonics [69,70,71,72]. Among the advantages of PIs and PBIs are their low-cost and commercially available starting material, rigid and planar aromatic scaffolds, extraordinary thermal and photochemical stability, appreciable and tunable visible light absorption, unique self-assembly behaviors [59,73,74,75,76], and low-lying FMO (frontier molecular orbitals) [59,76,77]. Due to their favorable combination of unusually high thermal and photostability, high molar extinction coefficients and fluorescence quantum yield, and excellent electron-transporting ability [78,79], PBIs have found applications in the dye industry (both as soluble dyes and insoluble pigments) [16,49] and organic optoelectronic devices (as n-type semiconductor materials), such as organic thin-film transistors [80], organic solar cells [42,81,82,83,84], organic light-emitting diodes (OLEDs) [49,85,86,87,88], organic field-effect transistors (OFETs) [89,90], fluorescent emitters [91], and molecular wires and sensors [23,34,35], and they serve as building blocks for light-harvesting and artificial photosynthetic systems [38,39,50] and liquid crystals [59]. It should also be noted that PBIs lead the way in the development of high-performance electron-accepting materials as alternatives to fullerenes in organic photovoltaics [92,93,94,95,96,97]. Aromatic benzene rings (one or more) and *B*-, *N*-, *O*-, *S*-, and *Se*-heterocycle core-extended (core-fused) derivatives [42,52,73,74,75,76,98,99,100,101,102,103,104,105,106,107,108,109,110,111,112,113,114,115,116] occupy a very important place among perylene derivatives, including tetraesters and, in particular, PBIs. The extension of the perylene core can be realized in different positions of this structure, such as the bay region or both the ortho and bay positions. Such modifications of perylene, PBIs, and other PAHs lead to perylene homologs such as benzoperylene, naphthoperylene, coronene, bisanthene, and other polyaromatic derivatives. In view of the above, novel properties can be easily derived while maintaining the aforementioned benefits of PAHs (i.e., thermo- and photostability). For instance, the extension of the perylene-3,4,9,10-tetracarboxylic tetraethyl ester core with sterically demanding groups can form [4] helicene fragments that are able to induce LC (liquid crystalline state) mesophases and changes in the absorption spectrum [100,101]. Benzoperylene hexacarboxylate, obtained via the Diels–Alder cycloaddition of maleic anhydride to perylene tetracarboxylic acid tetraester, can produce an electrode material for high-performance organic lithium-ion batteries [117]. This transformation of the perylene (or another PAH) core via Diels–Alder cycloaddition facilitates the incorporation of more redox groups and extension of the π-system in one step. Core-extended azobenzene-annulated PBIs efficiently coordinate to *Ru* and *Ir* complexes, which exhibit new and interesting optical properties [118]. Conductive polymers that contain fused perylene bisimide (which can be considered benzoperylene derivatives) and bithiophene units can be used as electron acceptors for efficient all-polymer solar cells [119]. Ring fusion of a perylene bisimide acceptor with thiophene can yield efficient non-fullerene organic solar cells with a small voltage loss and high power conversion [107,108]. By comparing fused and non-fused SMAs (non-fullerene small-molecule acceptors), it has been shown that ring fusion beneficially affects the properties and performances of the acceptor material [107,108]. Chemical modifications of PAHs are crucial for tuning their electronic and optical properties, and enabling self-assembly [4,120,121,122]. As mentioned previously, modifications of the bare perylene core can be carried out in various positions: peri, ortho, bay, and both ortho and bay regions (Figure 1). Functionalization of the perylene structure is an excellent method for obtaining derivatives with improved properties (i.e., optical properties can be tuned from the near-ultraviolet to the near-infrared spectral region) [15,16,88,121,123]. Straightforward functionalization of PAHs and their derivatives, i.e., perylene bisimides, particularly in the bay region, fundamentally affects their electronic and optical properties [16,119,123].

Perylene bay region (one or both of them) modifications can be realized via a substitution reaction of hydrogen atoms without core extension or via a reaction with such extension. For the synthesis that results in the extension of perylene, one of the following methods is usually applied. The first is a multistep method and involves the bromination-Sonogashira or Suzuki–Miyaura coupling reaction with monosubstituted acetylenes, with subsequent cyclization of mono- or diethynyl derivatives (thermal, photochemical, or catalytic annulation) to benzoperylenes or coronenes [124,125,126]. For instance, coronene and benzoperylene derivatives, i.e., tetraethoxycarbonyl dinaphthocoronene and (diethoxycarbonylnaphtho)tetra-ethoxycarbonylbenzo[*ghi*]perylene, were obtained from tetraethoxycarbonyl perylene according to the following sequence: bromination, followed by a Suzuki–Miyaura cross-coupling reaction with an arylboronic ester and, finally, photochemical cyclization with iodine [127,128]. Analogously, perylene-bisimide-helicene synthesis from perylene bisimides consists of the following steps: bromination, Suzuki–Miyaura coupling with a boronic ester, and cyclization (photochemical, with iodine) [129]. A perylene bisimide tetramer with a thiophene ring-fused structure was obtained in a similar manner: double Suzuki–Miyaura coupling and subsequent oxidative aromatization [107]. In turn, core expansion of perylene bisimide to bis-naphthocoronenebisimide was realized through the dibromination of PBI and then the *Pd*-catalyzed reaction of in situ-generated 2,3-naphthyne with dibromo-PBI [130]. A core-extended perylene derivative, dithieno[5,6-*b*;11,12-*b*’]coronene-2,3.8,9-tetracarbo-xylic tetraester, was prepared via the dibromination of a perylenetetracarboxylic tetraester, Stille-coupling of the dibromo derivative with 2-(tributylstannyl)thiophene, and, finally, cyclization in the presence of iodine [109,110]. The second one-step method of synthesizing bay-extended perylenes and other PAHs is realized through Diels–Alder cycloaddition to bay regions (both or, more frequently, one of them); in this approach, perylene or its derivatives and a wide range of dienophiles (maleic anhydride and imides, arynes, acetylenes, acetylenedicarboxylates, quinones, and others) are employed to yield the corresponding [4 + 2] adducts. Different routes of cycloadduct aromatization are possible (hydrogen evolution, oxidative dehydrogenation, and use of a nucleophile as a hydrogen acceptor). This type of annulative π-extension (APEX) strategy has emerged as a powerful and efficient synthetic method for the construction of polycyclic aromatic hydrocarbons and their functionalized derivatives, nanographenes, and π-extended heteroarenes. In contrast to other multistep syntheses that require perylene substrate prefunctionalization, APEX reactions minimize the number of preparative steps required to obtain complex structures [37,131,132,133,134]. It is worth mentioning that Diels–Alder cycloaddition to the bay region of perylene and its derivatives is a type of single-step APEX reaction and achieves the maximum synthetic efficiency in the construction of π-extended PAHs, including functionalized ones. This type of APEX was initially reported by Clar in 1932 with the reaction of perylene with maleic anhydride, which was then modified by Clar and Zander for the syntheses of benzoperylene and coronene. This field of chemistry was inactive for some time, but since 2009, when Scott and Fort published their results, it has been intensively developed [37,131,132,133,134]. Some Diels–Alder cycloadditions to the bay regions of perylene and its derivatives have been recently presented in a very general manner in reviews devoted to the synthesis of polycyclic aromatic compounds [1,4,37,131,132,133,134].

Herein, we provide an overview of all articles and patents devoted to the bay-region functionalization of perylene and its derivatives via Diels–Alder cycloaddition reactions using various dienophiles. This issue is discussed mainly from the perspective of organic synthesis, supplemented with theoretical calculations (in silico chemistry).

## 2. Cycloaddition to Unsubstituted Perylene

### 2.1. Cycloaddition of Maleic Anhydride

**Scheme 1:**
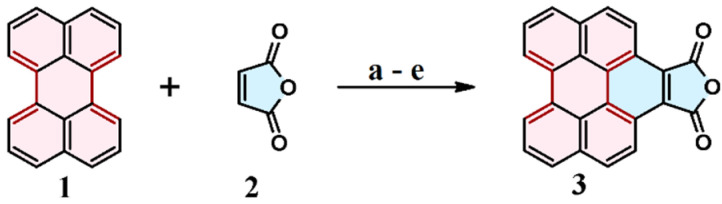
Cycloaddition of maleic anhydride to the perylene bay region. Reagents and conditions: (a) PhNO_2_, rfx, 1–1.5 h, Y = 45% [135]; (b) *p*-chloranil, rfx, 10 min, Y > 97% [136,137,138,139,140]; (c) *p*-chloranil, rfx, 10 min (oil bath at 240 °C), Y = 100% [126,141,142]; (d) *p*-chloranil, PhNO_2_, 190–200 °C, 20 min [143]; and (e) *p*-chloranil, CHCl_3_, 140 °C, 24 h, Y > 99% [144].

Maleic anhydride (**2**) is the first example of a dienophile that can be used in a Diels–Alder cycloaddition reaction to the bay position of the perylene molecule (**1**). Such a synthesis was described for the first time in 1932 by Clar. The reaction between perylene (**1**) and maleic anhydride (**2**) was carried out in the presence of nitrobenzene (which acted as a solvent and oxidizing agent) at reflux (202 °C) for 1–1.5 h, and it produced the expected product (**3**) in 45% yield [135]. An analogous reaction using molten maleic anhydride as the solvent (at reflux), with *p*-chloranil added to aromatize the new ring, and reduced time (10 min) resulted in a much higher yield (97%) [136,137]. This cycloaddition is thermally reversible, so using an oxidation agent (*p*-chloranil) is necessary to obtain aromatic products. *p*-Chloranil is most frequently used (nitrobenzene is also used but much less often), and the process of aromatization is irreversible, as shown in Scheme 1.

The cycloaddition of **2** to unsubstituted perylene (**1**) has been described many times. There are a few procedures with different reaction conditions, oxidizing agents, methods of product separation, and final yields. The details of the procedures can be found in the description under Scheme 1. The procedure described in 2014 [138] merits special attention because the reaction was performed in the presence of a small amount of chloroform solution from 140 °C to b.p., and the yield was almost quantitative. It should be added that the product of this reaction, i.e., benzo[*ghi*]perylene-1,2-dicarboxylic anhydride (**3**), is practically insoluble in typical organic solvents. 

**Scheme 2:**
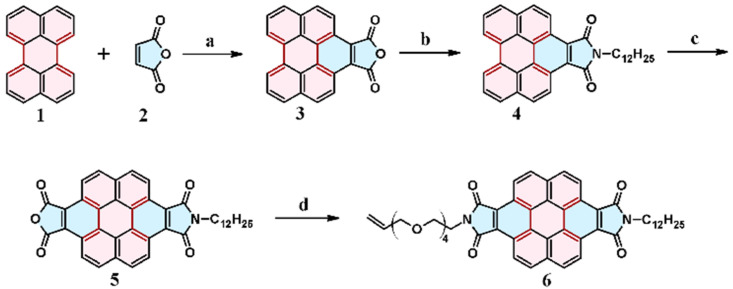
Synthesis of a coronene bisimide amphiphile using Diels–Alder cycloaddition to the bay region of perylene [126]. Reagents and conditions*:* (a) *p*-chloranil, 240 °C, 10 min, Y = 99%; (b) dodecylamine, DMF, rfx, 12 h, Y = 68%; (c) maleic anhydride, *p*-chloranil, 240 °C, 4 days, Y = not reported; and (d) glycolamine, DMF, rfx, 12 h, Y = 42%.

However, this relatively simple access to benzoperylene dicarboxylic anhydride (**3**) has opened up new pathways to the fabrication of the family of coronene bisimides, synthesized in the following cascade: Diels–Alder (DA) cycloaddition of maleic anhydride (**2**) to perylene (**1**) and the subsequent reaction of the obtained product (**3**) with the corresponding amine (the cascade repeats twice and, in the following steps, products **4**, **5**, and **6** were obtained), which is depicted in Scheme 2. It is worth mentioning that the final product of these reactions, i.e., **6** (*n*-type coronene bisimide amphiphile), is very soluble and exhibits varied optical properties, which can be controlled by the composition of the solvent.

In a similar reaction cascade, *N*-substituted benzo[*ghi*]perylenes with the following substituents were also obtained: isopropyl (yield not reported), nonan-5-yl (Y = 89%), undecan-6-yl (Y = 86%), and tridecan-7-yl (Y = 76%) [138].

### 2.2. Cycloaddition of N-Substituted Maleimides

**Scheme 3:**
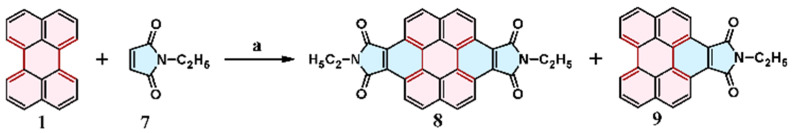
Cycloaddition of *N*-ethylmaleimide to the perylene bay region [141]. Reagents and conditions: (a) *p*-chloranil, *p*-hydroxyanisole, rfx (oil bath at 240 °C), 6 h, Y = 50%.

The cycloaddition of *N*-substituted maleimides to the perylene (**1**) bay region is also possible and even easier than the cycloaddition of maleic anhydride (**2**) because a double adduct (**8**) is obtained, as in Scheme 3. Similar to the product of maleic anhydride cycloaddition, the products of *N*-ethyl maleimide (**7**) cycloaddition are insoluble in all common solvents [126,141]. In the reaction shown below (Scheme 3), the monoadduct (**9**) was not separated.

**Scheme 4:**
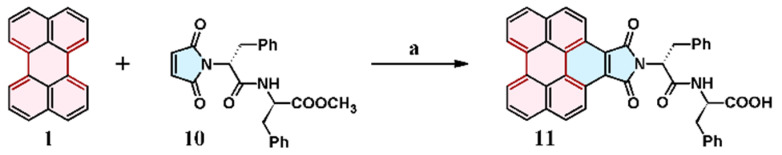
Cycloaddition of a maleimide derivative to perylene [47,48]. Reagents and conditions: (a) *p*-chloranil, 240 °C, 3 h, hydrolysis of COOMe to COOH; after DA cycloaddition, Y = 80%.

In 2014, Verma and coworkers described the cycloaddition of a chiral derivative of maleimide (**10**) to perylene (**1**), yielding *N*-maleoyl-D-Phe-D-Phe-OMe (94.3% yield); however, the product itself was not isolated. The monoadduct was converted into benzo[*ghi*]perylene-1,2-dicarboxylic(D-Phe-D-Phe-OH) (**11**) through the hydrolysis of the COOMe into the COOH group using LiOH, resulting in 80% yield (Scheme 4) [47,48].

An analogous reaction was reported two years later, in 2016, also by Verma’s research group. The final product (BPI-FF-OH) was used to synthesize BPI-FF-OH/cadmium-doped ZnO nanostructured lamellar hybrids [48].

### 2.3. Cycloaddition of Alkyl Acrylates

**Scheme 5:**
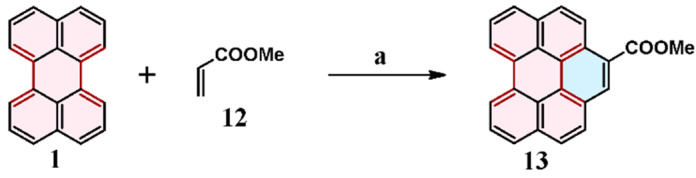
Cycloaddition of methyl acrylate to perylene [142]. Reagents and conditions: (a) *p*-chloranil, *p*-hydroxyanisole, 220 °C, 4 d, Y = 39%.

The application of alkyl acrylates as dienophiles in cycloadditions to the perylene core is also reported in the literature. Using appropriate acrylate dienophiles, Hirayama et al. synthesized benzo[*ghi*]perylene and coronene derivatives with electron-withdrawing methoxycarbonyl (COOMe) and methoxy (OMe) groups. Methyl benzo[*ghi*]perylene-1-carboxylate (**13**) was synthesized via the reaction of methyl acrylate (**12**), which also acted as a solvent, with **1** in the presence of *p*-chloranil (used for cycloadduct aromatization) and *p*-hydroxyanisole (acting as a radical scavenger, which limits the polymerization of acrylate), as illustrated in Scheme 5 [142].

The further reaction between the obtained cycloadduct, i.e., 1-methoxybenzo[*ghi*]perylene (**13**), and maleic anhydride (**2**) in the presence of *p*-chloranil results in coronene derivatives (see Section 9) [126].

**Scheme 6:**
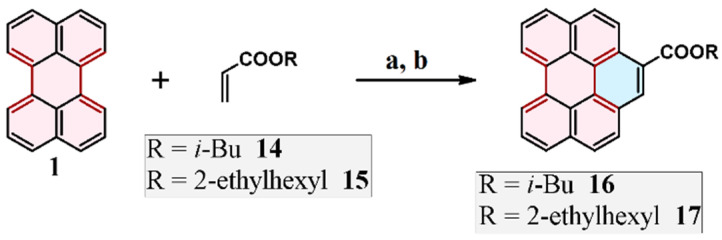
Cycloaddition of isobutyl or 2-ethylhexyl acrylate to perylene [143]. Reagents and conditions: (a) for R = *i*-Bu: *p*-chloranil, *p*-hydroxyanisole, rfx (132 °C), 6 d, Y = 39% and (b) for R = 2-ethylhexyl: *p*-chloranil, *p*-hydroxyanisole, rfx (215 °C), overnight, Y = 41%.

The synthesis of other benzoperylene derivatives, namely, benzo[*ghi*]perylene-1-carboxylic-(isobutyl)ester (**16**) and benzo[*ghi*]perylene-1-carboxylic-(2-ethylhexyl)ester (**17**), has also been reported. The first product can be obtained via the cycloaddition reaction of isobutyl acrylate (**14**) with perylene (**1**) at reflux (132 °C) in the presence of *p*-chloranil and *p*-hydroxyanisole, which is depicted in Scheme 6. Unfortunately, the yield of this reaction is low (39%), and the perylene conversion is only 64%. When using a higher boiling acrylate (**15**) as a dienophile, the conversion of perylene is almost quantitative. Nevertheless, because of the difficulty in separating the pure product of the reaction, the isolated yield is also low (41%), as reported in Scheme 6. 

It is worth mentioning that alkyl acrylates are much less reactive than maleic anhydride: in the case of the latter, the reaction is complete after a few minutes at reflux (ca. 202 °C) [141].

### 2.4. Cycloaddition of Dialkyl Diazenedicarboxylate

**Scheme 7:**
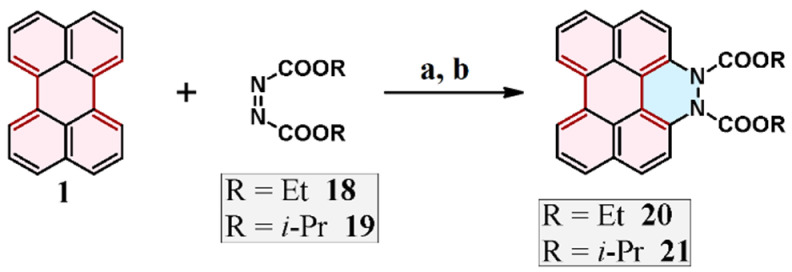
Synthesis of 1,2-diazobenzo[*ghi*]perylene derivative via the cycloaddition of dialkyl diazene dicarboxylates to the perylene bay region [145]. Reagents and conditions: (a) for R = Et: 160 °C, 1.5 h, Y = 40% and (b) for R = *i*-Pr: 160 °C, 4.5 h, Y = 32%.

Tokita et al. described the cycloaddition of dialkyl diazenedicarboxylates to the bay regions of perylene (**1**), dibenzo[*b*,*n*]perylene (**199**) (see Section 9), and 1,2-diazobenzo[*ghi*]perylene (see Section 9), as shown in Scheme 7 [145]. The products of these cycloaddition–aromatization reactions are the corresponding PAHs and hydrazine-*N*,*N*’-dicarboxylates (the latter are the result of hydrogen transfer from the cycloadduct to dienophile products). Therefore, the reaction discussed here proceeds somewhat differently than the cycloaddition–rearomatization of dialkyl acetylenedicarboxylates to **1**, in which hydrogen elimination takes place. Furthermore, in the case of the cycloaddition–rearomatization reaction, hydrogen transfer to the dienophile is not observed (dialkyl maleate or fumarate was not detected) [145]. In the earlier-mentioned publication, diethyl (**18**), diisopropyl (**19**), and di-*t*-butyl diazenedicarboxylates were used as dienophiles. Reactions with the first two diazenedicarboxylates produce **20** and **21** in 40% and 32% yields, respectively.

**Scheme 8:**
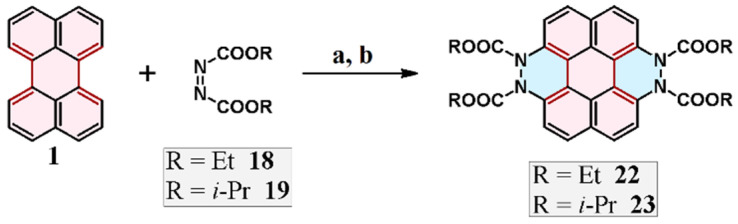
Cycloaddition of diazenedicarboxylates to both bay regions of the perylene molecule [145]. Reagents and conditions: (a) for R = Et: 160 °C, 6 h, Y = 38% and (b) for R = *i*-Pr: 160 °C, 12 h, Y = 27%.

Cycloaddition to both bay regions is also possible; however, it is necessary to prolong the time of heating (Scheme 8) [145]. Products **22** and **23** were formed in 38% and 27% yields for R = Et and R = *i*-Pr, respectively. Unfortunately, the reaction in which di-*t*-butyl diazenedicarboxylate was used as a dienophile did not result in the expected product (even when the heating time was extended), perhaps because of its great steric hindrance.

### 2.5. Cycloaddition of Benzo- and Naphthoquinones

**Scheme 9:**
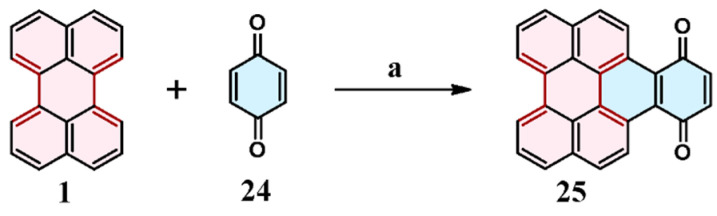
Cycloaddition of 1,4-benzoquinone to the perylene bay region [146]. Reagents and conditions: (a) PhNO_2_, rfx, 1.5 h, Y = 36.5%.

In 1968, the cycloaddition of *p*-benzoquinone (**24**) to perylene (**1**), leading to naphtho[*ghi*]perylene-1,4-dione 25, was described (Scheme 9) [146]. Nitrobenzene acted as a mild oxidizing agent. The obtained product (**25**) was subjected to a reduction reaction (red phosphorus, HI, 220 °C, 60 h), which resulted in naphtho[*ghi*]perylene.

**Scheme 10:**
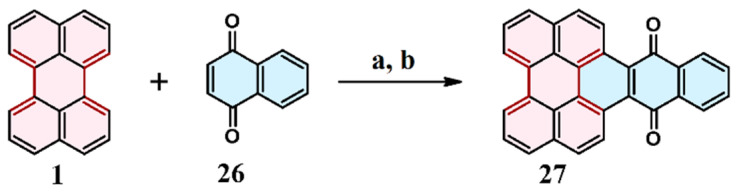
Cycloaddition of 1,4-naphthoquinone to the perylene bay region [146]. Reagents and conditions: (a) *p*-chloranil, PhNO_2_, rfx, 6 h and (b) PhNO_2_, rfx, 16.5 h; Y = 70%.

In the same work, the reaction between perylene (**1**) and 1,4-naphthoquinone (**26**), resulting in **27**, was shown (Scheme 10). This reaction was realized using *p*-chloranil as an oxidizing agent. However, it turns out that the application of this oxidizing agent is not necessary: the reaction can be carried out using only nitrobenzene, which acts both as a solvent and an oxidant [146].

Similar to the reaction shown in Scheme 9, in the reaction of perylene (**1**) with 1,4-naphthoquinone (**26**), the cycloaddition product, i.e., **27**, was also reduced to an acene, namely, anthracenoperylene, using zinc powder, NaCl, and ZnCl_2_ at 240–250 °C, which results in a color change from brown to olive-green, and then 290–295 °C [146]. No diadduct was found in either reaction. 

### 2.6. Cycloaddition of Acetylene

**Scheme 11:**
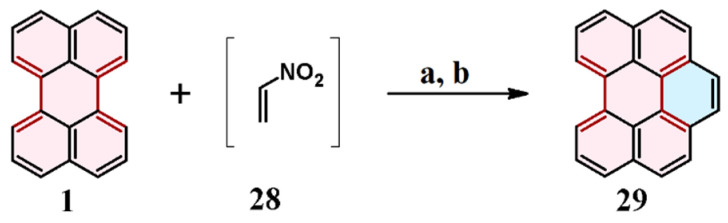
Synthesis of benzo[*ghi*]perylene via the cycloaddition of acetylene (in situ generated from nitroethene) [147,148]. Reagents and conditions: (a) 2-nitroethanol (acetylene source), phthalic anhydride, pressure vessel, 175 °C, *o*-Cl_2_C_6_H_4_, 4 d, Y = 58% [147] and (b) 2-nitroethanol (acetylene source), phthalic anhydride, 200 °C, MW, o-Cl_2_C_6_H_4_, 20 h, mixture of product and starting material was obtained (ratio 1.3:1, Y = 55%) [148].

In 2010, Fort and Scott synthesized benzo[*ghi*]perylene (**29**) via the cycloaddition of perylene (**1**) with acetylene, which was generated in situ from nitroethanol as masked acetylene (Scheme 11) [147]. The authors suggested that the mechanism of this reaction occurs in three distinct steps: cycloaddition of nitroethene (**28**) to the perylene core, thermal elimination of a hydrogen molecule, and, finally, thermal elimination of HONO [147].

In 2016, Jackson et al., using the same substrates (**1** and **28**), carried out an analogous cycloaddition, but the reaction conditions were changed. Using microwaves, they obtained a mixture of the product and starting material with a 1.3:1 ratio and 55% yield (see Scheme 11) [148]. It should also be pointed out that the cycloaddition of nitroethylene to perylene fails if the in situ generation protocol is not employed [147]. This cycloaddition of acetylene (or masked acetylene) to the bay regions of PAHs, resulting in a non-substituted benzene ring, can be applied in a carbon nanotube growing strategy via DA cycloaddition–rearomatization reactions [45].

**Scheme 12:**
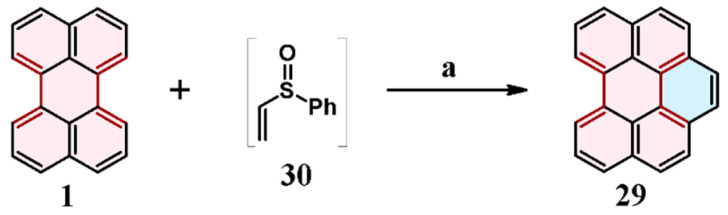
Synthesis of benzo[*ghi*]perylene via the cycloaddition of phenyl vinyl sulfoxide to the perylene bay region [147]. Reagents and conditions: (a) pressure vessel, 155 °C, 4 d, Y = 8%.

The cycloaddition of phenyl vinyl sulfoxide (**30**), applied as masked acetylene, to perylene (**1**) also resulted in benzo[*ghi*]perylene (**29**) (Scheme 12) [147]. Unfortunately, the reaction had a very poor yield (<10% after 4 days). Moreover, the prolonged reaction time caused the polymerization and decomposition of the dienophile.

Comparing the reactivity of nitroethylene (**28**) and phenyl vinyl sulfoxide (**30**) in Diels–Alder cycloaddition reactions, it can be inferred that the first one is a much better dienophile. Phenyl vinyl sulfoxide (**30**) polymerizes/decomposes very quickly in the reaction not only with perylene but also with the much more reactive bisanthene (this cycloaddition takes place but is much less straightforward and efficient than with nitroethanol as a dienophile; also see Section 11.2). Furthermore, it is known that selenoxide is an even worse dienophile than sulfoxide [147]. The differences in the reactivity of dienophiles, which can be applied as “masked acetylene”, e.g., QCH=CH2 (Q = PhS(O)-, O_2_N-, F_5_C_6_-, F_3_C-), are also confirmed by theoretical calculations (see Section 12).

### 2.7. Cycloaddition of Acetylenedicarboxylates

**Scheme 13:**
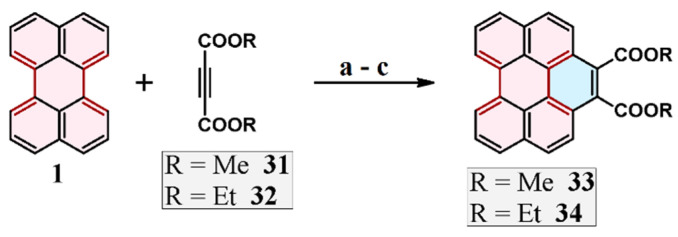
Cycloaddition of acetylene dicarboxylates to the perylene bay region [143,149,152,153]. Reagents and conditions: (a) for R = Me: *p*-chloranil, PhNO_2_, 200 °C, 1 h, Y = 22%; (b) for R = Et: toluene, 150 °C, 3 d, Y = 25%; and (c) *p*-cymene, 185 °C, pressure vessel, 24 h, Y = 95% (**33**); Y = 96% (**34**).

The cycloaddition of acetylenedicarboxylates to the perylene (**1**) core was described in 1959 by Hoppf and Schweizer. The reaction between **1** and dimethyl acetylenedicarboxylate (**31**) proceeded in nitrobenzene in the presence of *p*-chloranil and resulted in 22% yield [143]. In 2009, cycloaddition reactions of diethyl acetylenedicarboxylate (**32**) to the bay region of perylene (**1**) and bisanthene were described (see Section 11.3), as shown in Scheme 13 [149]. As a byproduct of this reaction, hydrogen is released, and, importantly, acetylenedicarboxylate does not act as a hydrogen acceptor [149,150,151]. Significantly, in contrast to the reaction with dimesitylbisanthene (**208**) (see Section 11.3), cycloaddition to both bay regions was not observed, so coronene derivatives were not formed.

DFT (Density Functional Theory) calculations were also carried out for a better understanding of significant differences in reactivity between perylene and 7,14-dimesitylbisanthene (the latter is much more reactive), as discussed in Section 12.

Recently, we showed that raising the temperature of this reaction (perylene with acetylenedicarboxylates) to 185 °C (in *p*-cymene and a pressure vessel) allows the quantitative conversion of **1** and high isolated yield of benzo[*ghi*]perylene derivatives (Y = 95% and 96% for **33** and **34**, respectively) after 24 h of heating [152,153].

### 2.8. Cycloaddition of Diarylacetylenes

**Scheme 14:**
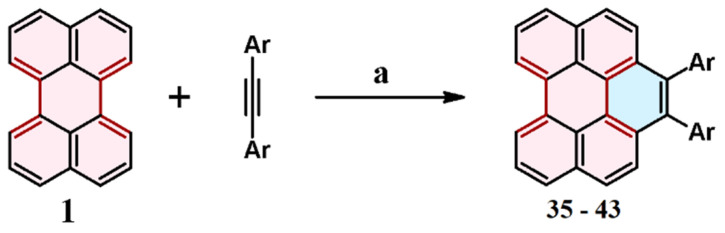
Cycloaddition of diarylacetylenes to the perylene bay region: synthesis of 1,2-diarylbenzo[*ghi*]perylenes [152,154,155]. Reagents and conditions: (a) 10 eq. of perylene, solvent (Ph_2_O) or excess of melted perylene, 280–300 °C, 0.001 Pa, 72 h, Y = up to 50%; Ar = see Figure 2.

**Figure 2:**
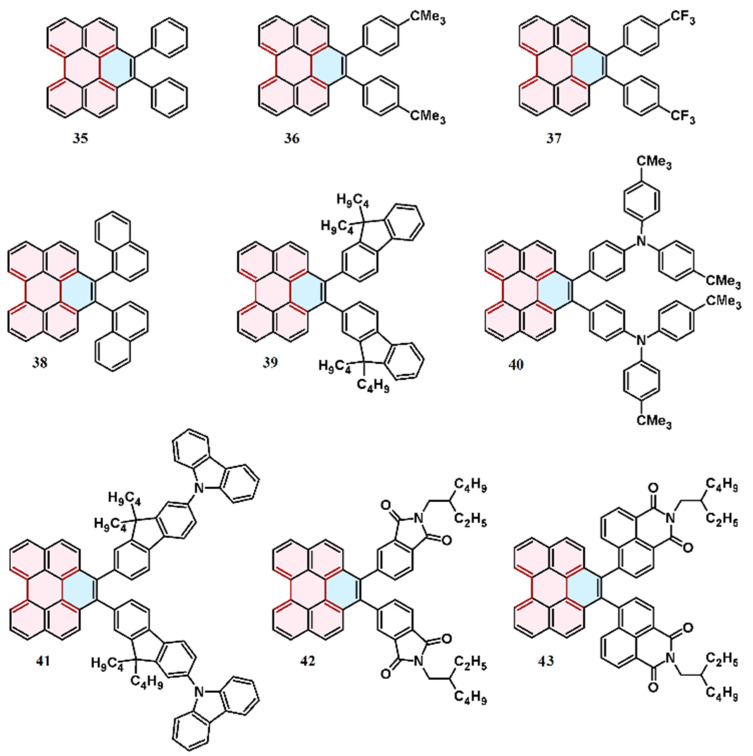
Cycloaddition of 1,2-diarylacetylenes to the perylene bay region: list of obtained products. Y = 45% (**35**), 18% (**36**), 27% (**37**), 7% (**38**), 50% (**39**), 21% (**40**), 33% (**41**), 42% (**42**), and 10% (**43**).

Recently, our group’s research showed that the cycloaddition of different diarylacetylenes to the perylene (**1**) bay region to afford the corresponding 1,2-diarylbenzo[*ghi*]perylenes (**35**-**43**) is also possible, despite the high activation energy (also see Section 12) calculated for the model reaction of diphenylacetylene to perylene (see Scheme 14 and Figure 2) [152,154,155].

The above-mentioned reaction creates completely new possibilities for functionalizing the perylene core and synthesizing novel precursors of smaller graphene pieces. Undoubtedly, the big advantage of this reaction is the use of an excess of molten perylene, which can be quantitatively removed via sublimation [152,155]. The reaction conditions (especially temperature) were selected on the basis of computational calculations (see Section 12). 

### 2.9. Reaction of 1,4-Diaryl-1,3-Butadiynes with Perylene: Domino-Type Cycloaddition–Cycloaromatization

**Scheme 15:**
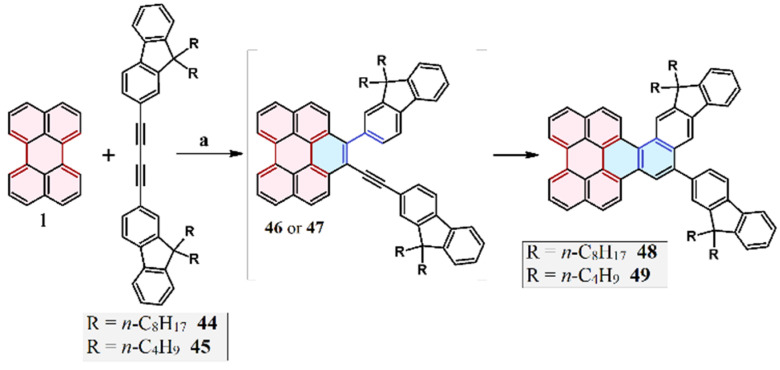
From perylene and diarylbutadiynes to π-extended PAHs via tandem DA cycloaddition–cycloisomerization [152,165,166]. Reagents and conditions: (a) 10 eq. of diyne, 280 °C, 72 h, <0.01 Pa, Y = 10% (**48**) and 8% (**49**).

Recently, we discovered a brand-new reaction of perylene **1** with 1,4-diaryl-1,3-butadiynes (**44** and **45**) that leads to π-extended perylene derivatives (**48**-**49**), as shown in Scheme 15. The results of DFT calculations were our inspiration, especially because the activation energy of the cycloaddition of PhC≡C-C≡CPh to perylene was lower than that of PhC≡CPh (see Section 12). In the first stage of this reaction, the [4 + 2] cycloaddition of diarylbutadiyne (**44** or **45**) to the **1** bay region takes place. Importantly, diarylbutadiyne reacts analogously to disubstituted acetylene, and only one of the triple bonds undergoes cycloaddition. In the second stage, the resulting aryl-(arylethynyl)benzo[*ghi*]perylene (**46** or **47**) rapidly undergoes a cycloaromatization reaction, similarly to masked dienyne, and results in **48** or **49**, which is well-documented [156,157,158,159,160,161,162,163,164,165,166]. Thus, it is a domino-type reaction: Diels–Alder cycloaddition with subsequent cycloaromatization [152,165,166].

The cycloisomerization (cycloaromatization) of compounds, which are structurally similar to the intermediates (**46** or **47**) formed in the reaction shown in Scheme 15, is described in the literature. It is based on transformation using ethynylarenes and conjugated dienynes, including masked ones [156,157,158,159,160,161,162,163,164,165,166].

### 2.10. Cycloaddition of Benzyne and Naphthynes

**Scheme 16:**
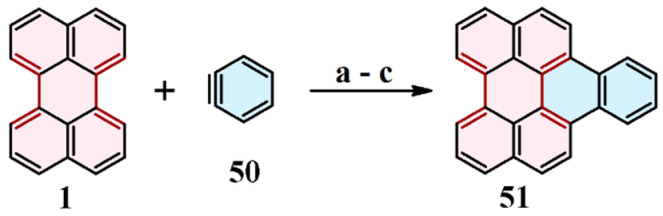
Cycloaddition of benzyne to the perylene bay region: synthesis of naphtho[*ghi*]perylene [167,168,169]. Reagents and conditions: (a) benzenediazonium 2-carboxylate, DCM/THF (4*v*/1*v*), rfx, 3.5 h, Y = 66%; (b) NBu_4_F trihydrate (TBAF), 2-(trimethylsilyl)phenyl trifluoromethanesulfonate (benzyne precursor), PhMe, rfx, 2.5 h, Y = 45%; and (c) phthalic anhydride (benzyne precursor), high-temperature flow system (up to 1000 °C), Y = 4.8%.

The cycloaddition reaction to the perylene core using arynes as dienophiles was described for the first time in 1966 by Stork and Matsuda in a U.S. patent. The authors proposed a method to generate benzyne (**50**) from benzenediazonium 2-carboxylate, which is created in situ from anthranilic acid and isoamyl nitrite. The final product of the generated benzyne and perylene (**1**), namely, naphtho[*ghi*]perylene (**51**), was obtained in 66% yield (see Scheme 16) [167]. Recently, a new variant of the above-described benzyne-generation method was reported. First, a diazonium salt is synthesized, and then it is subjected to a reaction with KF and 18-Crown-6 to generate benzyne [168]. Thus-generated **50** undergoes in situ cycloaddition to **1**, and naphtho[*ghi*]perylene (**51**) is formed in 77% yield. Therefore, it is a two-stage process, which is a serious drawback. Moreover, if the yield of the synthesis of diazonium salt is taken into account, the final yield of **51** is about 10% lower.

In 2011, Fort and Scott published their studies dedicated to the cycloaddition of **50**, generated in the gas phase from phthalic anhydride or in solution from (2-trimethylsilyl)phenyl trifluoromethanesulfonate, to the perylene (**1**) bay region (Scheme 16). According to the authors, the reaction in the gas phase can be a useful strategy for the growth of carbon nanotubes [169]. 

**Scheme 17:**
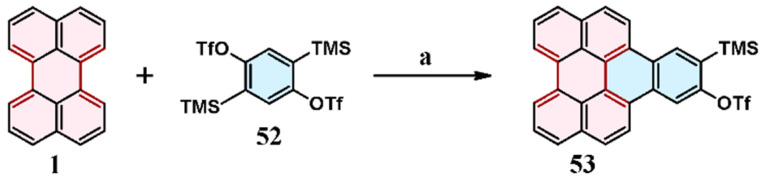
Monocycloaddition of benzo-diyne precursor to the perylene bay region [170]. Reagents and conditions: (a) CsF, ThF/MeCN (1*v*/1.8*v*), 60 °C, 16 h, Y = 49%.

In 2014, Schuler and his colleagues proposed the cycloaddition of bis-benzyne precursor **52** to perylene (**1**), leading to monoadduct (**53**) formation, wherein the conditions of this reaction, including the temperature and solvent, were selected to reduce the regeneration of benzyne, and, consequently, limited the formation of the double adduct (Scheme 17) [170].

**Scheme 18:**
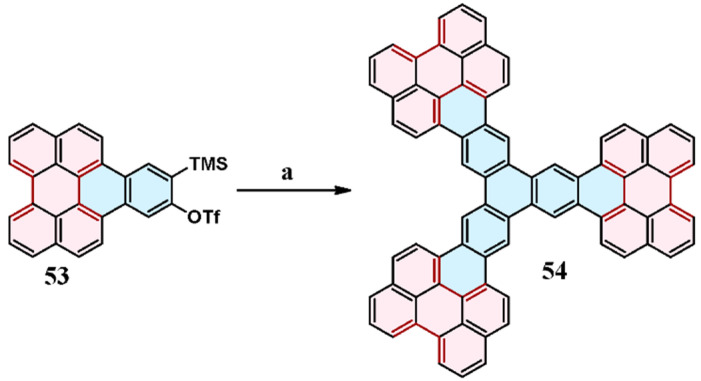
Cyclotrimerization of perylene-fused-benzyne to star-shaped-nanographene [170]. Reagents and conditions: (a) Pd_2_(dba)_3_, CsF, THF/MeCN (1*v*/1*v*), 60 °C, 16 h, Y = 46%.

Subsequently, the resulting perylene derivative (**53**) was subjected to the palladium-catalyzed [2 + 2 + 2] cyclotrimerization of an aryne using CsF and a catalytic amount of [Pd_2_(dba)_3_]. This reaction resulted in a star-shaped nanographene-like material (**54**), which is depicted in Scheme 18 [170]. However, the obtained nanographene (**54**) does not contain any substituents and is completely insoluble in organic solvents [170].

It should be added that there are a lot of publications devoted to the synthesis of various nanographenes in the literature, including star-shaped and functionalized ones from different arynes. For instance, the synthesis of soluble TIPS-ethynyl-starphenes via Yamamoto coupling–cyclotrimerization of TIPS-dibromo-tetra- or pentacene precursors was described in 2011 [171]. The cyclotrimerization of arynes to phenanthrene-fused-benzyne was also described [171]. Multiple examples of applications of benzyne, bis-benzyne, arynes, and bis- and polyarynes in the synthesis of PAHs, including functionalized ones, via cycloaddition, double-cycloaddition, cyclotrimerization, and other reactions are described in the literature [37,163,171,172,173,174].

**Scheme 19:**
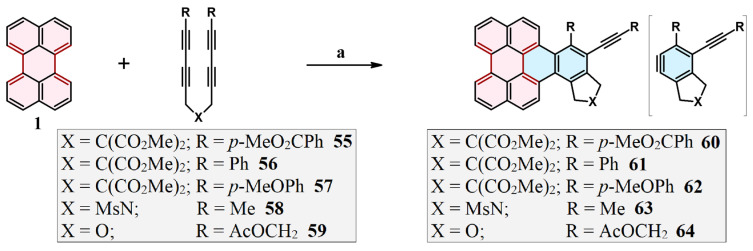
Synthesis of naphtho[*ghi*]perylene derivatives via HDDA and bay-region DA cycloaddiotion cascade reactions [175]. Reagents and conditions: (a) CHCl_3_, 85 °C, ~12 h, Y = 56% (**60**); 56% (**61**); 32% (**62**); 53% (**63**); and 31% (**64**).

Another reaction worth mentioning is the synthesis of naphtho[*ghi*]perylene derivatives (with the structures described by **60**-**64**) via the cycloaddition of benzyne derivatives to the bay region of **1** (HDDA reaction: hexadehydro-Diels–Alder reaction), which was described by Xu et al. in 2016 [175]. The benzyne derivatives were produced in situ by the thermal cycloisomerization of tetrayne substrates (**55**-**59**), as illustrated in Scheme 19. 

**Scheme 20:**
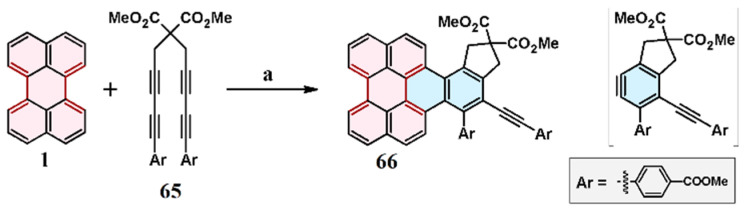
Synthesis of core-extended perylene via the cascade of photo-HDDA and DA to the bay region [176]. Reagents and conditions: (a) 1.5 eq. of perylene, quartz, 300 nm, 23 °C, CHCl_3_, Y = 65%.

An analogous reaction was also carried out for tetraetoxycarbonylperylene (see Section 8). In 2017, Hoye et al. demonstrated that the HDDA cyclotrimerization reaction to produce benzyne derivatives could be initiated photochemically using tetrayne **65** [176]. They presented the synthesis of core-extended PAH **66** via benzyne cycloaddition to the perylene bay region (see Scheme 20).

**Scheme 21:**
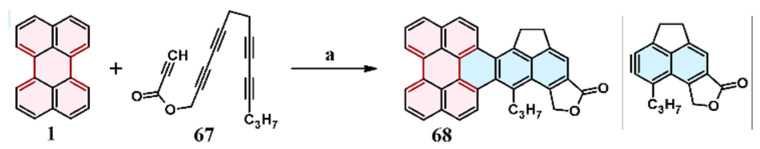
2,3-Naphthyne derivative cycloaddition to the perylene bay region [177]. Reagents and conditions: (a) 3 eq. of perylene, CHCl_3_, Y = 54%.

In 2018, Xiao and Hoye showed the development and use of the domino HDDA reaction to synthesize structurally diverse polyacenes from acyclic polyyne precursors [177]. The key factor in these transformations is the successive thermal reaction of multiple 1,3-butadiyne units, e.g., **67**, with a series of in-situ-generated diynophilic arynes to produce higher polyacynes. This method was applied to the synthesis of anthracene-fused-perylene (**68**) via the DA cycloaddition of an in-situ-generated naphthyne derivative to the perylene (**1**) bay region (Scheme 21).

**Scheme 22:**
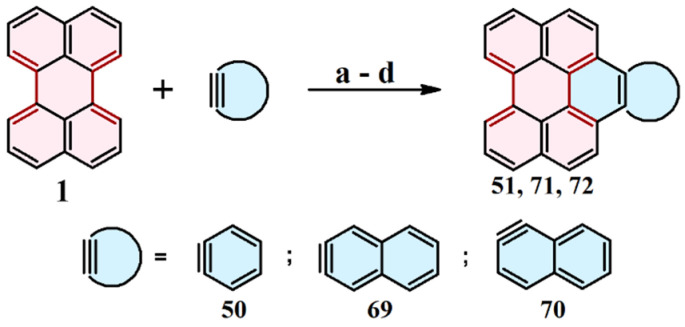
Cycloaddition of arynes to the perylene bay region [178,179,180,181]. Reagents and conditions: (a) 4 eq. of aryne precursor, CsF, MeCN/THF (1*v*/1*v*), Y = 87% (**51**), 64% (**71**), and 57% (**72**); (b) 8 eq. of aryne precursor, KF, 18-Crown-6 1.0 M solution in THF, THF, 60 °C, 72 h, Y = 85% (**51**), 66% (**71**), and 61% (**72**); (c) 10 eq. of benzyne precursor, KF, Bu_4_NF 1.0 M solution in THF, Y = 86% (**51**); and (d) 4 eq. of aryne precursor, CsF, succinonitrile/1,2-dimethoxyethane (1*v*/1*v*), 60 °C, 24–96 h; Y = 95% (**51**), 79% (**71**), and 70% (**72**).

Recently, our research group developed methods for the cycloaddition reaction of benzyne (**50**) and 1,2- and 2,3-naphthynes (**71** and **72**, respectively) to the **1** bay region, resulting in corresponding perylene derivatives **51**, **71**, and **72** (see Scheme 22) [178,179,180,181]. We tested four aryne-generation methods for the reaction of perylene with benzyne generated from 2-trimethylsilyl triflate, and all of them proved to be effective, furnishing the expected product (**51**) with high yields. In the case of benzyne generation using the system KF + Bu4NF in THF, adding a large excess of KF and lowering the temperature to 60 °C turned out to be crucial. The yield of the reaction increased significantly compared with that obtained by Fort and Scott [169].

The cycloaddition reactions of the arynes (**50**, **69**, and **70**) depicted in Scheme 22 were also realized for perylene tetracarboxylic acid tetrabenzyl ester (see Section 8). These reactions are even more effective when a mixture of 2-dicyanoethene and 1,2-dimethoxyethane, instead of MeCN and THF, is used. The efficiency of this procedure is particularly evident in the cycloaddition reactions of benzyne to diarylbenzoperylenes (see Section 9).

## 3. Cycloaddition to Alkyl Perylenes

### 3.1. Cycloaddition of Maleic Anhydride

**Scheme 23:**
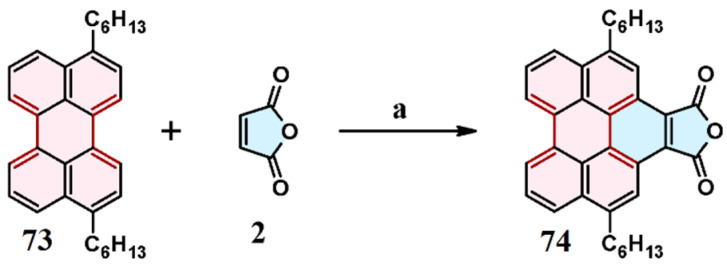
Cycloaddition of maleic anhydride to 3,10-di(*n*-hexyl)perylene [182]. Reagents and conditions: (a) *p*-chloranil, CHCl_3_, 140 °C (sealed tube), 96 h, Y = 16%.

The cycloaddition of maleic anhydride (**2**) to 3,10-di(*n*-hexyl)perylene (**73**) was described by Cammidge and Gopee (Scheme 23) [182]. Despite the presence of two alkyl groups, the final product of this reaction (**74**) was only sparingly soluble in common solvents and hence difficult to purify and handle. Moreover, the reaction yield was low—only 16% (significantly lower than the near-quantitative yield for unsubstituted perylene (**1**))—and degradation products were observed. 

### 3.2. Cycloaddition of Fumarodinitrile to 3,10-di-(N-Hexyl)- and 3,10-di-(N-Dodecyl)Perylene

**Scheme 24:**
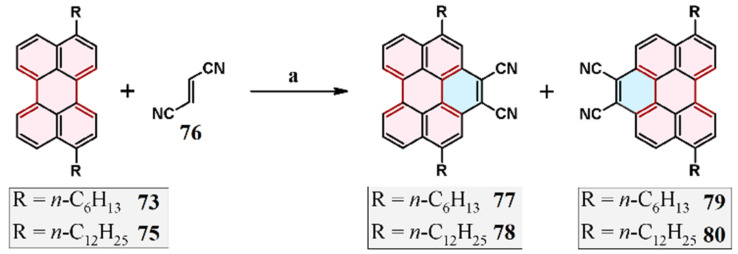
Cycloaddition of fumaronitrile to 3,10-di(*n*-hexyl)- and 3,10-di(*n*-dodecyl)perylene [182]. Reagents and conditions: (a) *p*-chloranil, CHCl_3_, 140 °C (sealed tube), 96 h.

Cammidge and Gopee also presented the cycloaddition of fumarodinitrile (**76**) to the bay regions of 3,10-dialkylperylenes (**73** and **75**), which is shown in Scheme 24 [182].

As a result of this reaction, two regioisomers (**77** and **78** for **73**; **79** and **80** for **75**) were formed. Unfortunately, because of the formation of degradation products, the yields were extremely low (2% + 5% for **77** and **78**; 1% + 0% for **79** and **80**, respectively) [182].

### 3.3. Cycloaddition of Triazolinedione

**Scheme 25:**
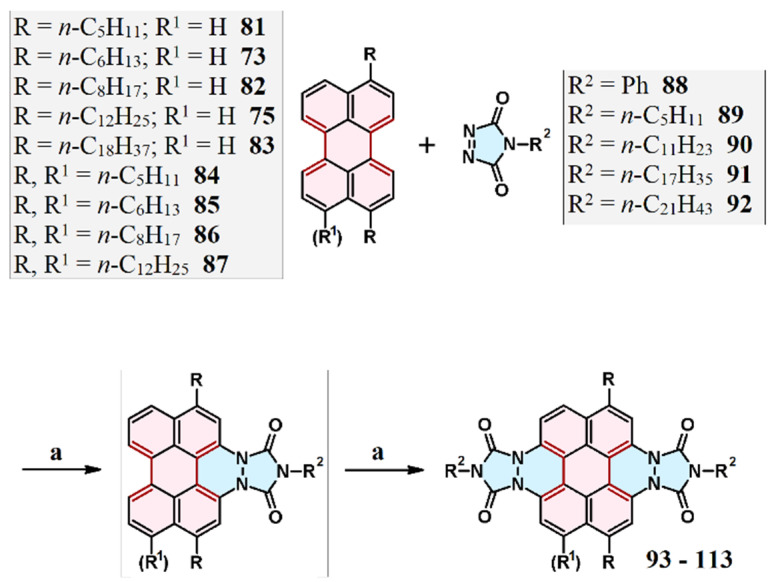
Synthesis of 1,2,6,7-tetraazocoronenes via the cycloaddition of 4-(*n*-alkyl)-1,2,4-triazol-3,5-dione to 3,(9 and10)-dialkyl perylenes [183,184]. Reagents and conditions: (a) for all combinations of **75**, **81**, and **83** with **88**-**92**: xylene, rfx, Y = 82% (for the reaction of **81** with **88**; yields for other substituents were not reported) [183] and (b) for the cycloaddition of **73**, **75**, **81**, **82**, and **84**-**87** with **91**: xylene, rfx; Y = up to 95% [184].

In 1993 [183] and then again in 1998 [184], the cycloaddition reaction between 4-(*n*-alkyl)-1,2,4-triazol-3,5-diones (**88**-**92**) and 3,10- and 3,9-dialkylperylenes (**73**,**75**,**81**–**87**), producing 3,9(10)-di(*n*-alkyl)-5,6.11,12-tetraazo-5,6,11,12-tetrahydrocoronene-5,6,11,12-tetracarboxylic acid di-*N*-substituted-bisimides (**93**-**113**), was reported (Scheme 25) [183,184].

The reaction mixture was heated until the starting material (yellow) and monoadduct (red) completely vanished. Diadducts (**93**–**113**) were obtained in high yields, but monoadducts were not isolated. Importantly, *p*-chloranil was not necessary in this reaction. The reaction depicted in Scheme 25 is an example of the cycloaddition of triazolinediones to the bay regions of both perylene and 1,2-diazobenzo[*ghi*]perylene (also see Section 7.3).

### 3.4. Cycloaddition of Dialkyl Acetylenedicarboxylates

**Scheme 26:**
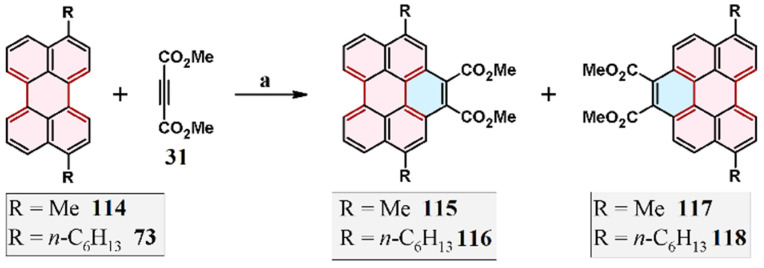
Cycloaddition of dimethyl acetylenedicarboxylate to 3,10-dimethyl- and 3,10-dihexylperylene [182]. Reagents and conditions: (a) *p*-chloranil, PhNO_2_, rfx, 2 h; R = Me (45% overall yield; 115:117 = 3:1); R = n-C_6_H_13_ (15% overall yield; 116:118 = 2:1).

In 2006, DA cycloaddition reactions of dimethyl acetylenedicarboxylate (**31**) to 3,10-dimethyl- and 3,10-di-(*n*-hexyl)perylene (**114** and **73**, respectively) were reported (Scheme 26) [182].

The conversions of both dialkylperylenes **73** and **114** were quantitative. As a result of these reactions, two possible regioisomers were formed (**115** and **117** for **114**; **116** and **118** for **73**), but the reaction yield for the *n*-hexyl derivative was substantially lower than that for the methyl one (45% and 15%, respectively). Degradation products were formed, similar to other cycloadditions to dialkyl perylenes (see Section 3.1 and Section 3.2). It should be added that no publications devoted to cycloadditions to dialkyl perylenes discuss the reasons for such a formation. Furthermore, the creation of coronene derivatives (i.e., the substitution of perylene in both bay positions) was not observed. 

## 4. Cycloaddition to Nitroperylene

**Scheme 27:**
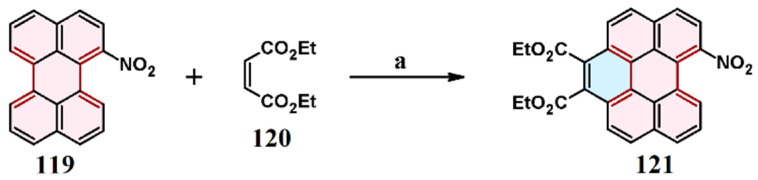
Synthesis of diethyl 7-nitrobenzo[*ghi*]perylene-1,2-dicarboxylate via cycloaddition/aromatization of diethyl maleate to the 1-nitroperylene bay region [185]. Reagents and conditions: (a) 180 °C, 5 h, then *p*-chloranil (after cooling to rt.), then 180 °C, 24 h; Y = 51%.

The cycloaddition of diethyl maleate (**120**) to the 1-nitroperylene (**119**) bay region, resulting in diethyl 7-nitrobenzo[*ghi*]perylene-1,2-dicarboxylate (**121**), as shown in Scheme 27, was described in 2008 by Jiang et al. [185].

The introduction of two carboxylate moieties significantly improves the solubility of final products and also affects the solid-state packing mode. In this reaction, it is necessary to employ *p*-chloranil as a dehydrogenation agent [185].

## 5. Cycloaddition to Dicyanoperylene

**Scheme 28:**
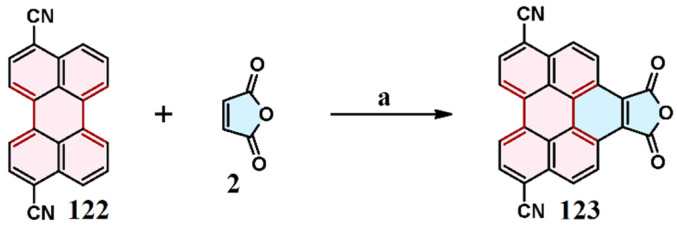
Synthesis of 5,10-dicyano-benzo[*ghi*]perylene-1,2-dicarboxylic anhydride via the cycloaddition of maleic anhydride to dicyanoperylene [186]. Reagents and conditions: (a) excess of molten maleic anhydride, *p*-chloranil, rfx, 24 h, Y = not reported.

Kelber et al. presented the cycloaddition reaction of maleic anhydride (**2**) to the dicyanoperylene (**122**) bay region (Scheme 28). However, the cycloadduct (**123**) itself was not isolated, as it was immediately converted into a diester, and the reaction resulted in an overall yield of 46% [186]. It should be noted that the cycloaddition was completely regioselective, and only one cycloadduct was obtained (see Scheme 28).

## 6. Cycloaddition to Aryl(diaryl)perylenes

**Scheme 29:**
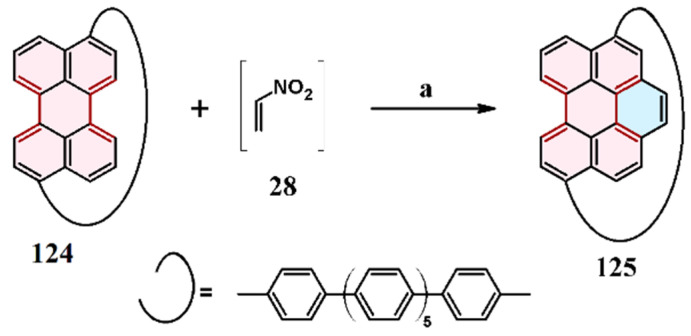
Cycloaddition of acetylene generated in situ from nitroethanol to a cycloparaphenylene containing a perylene motif [148]. Reagents and conditions: (a) 2-nitroethanol (acetylene source), phthalic anhydride, 200 °C, MW, *o*-Cl_2_C_6_H_4_, 20 h.

In 2016, Jackson and colleagues described the cycloaddition of acetylene, generated in situ from nitroethanol, to a cycloparaphenylene containing a perylene motif (**124**), as shown in Scheme 29 [148].

Unfortunately, all efforts to separate the cycloaddition product from the starting material were unsuccessful. The yield was determined with the aid of NMR and MALDI-TOF spectra.

## 7. Cycloaddition to Perylene Bisimides and Their Derivatives

### 7.1. Cycloaddition of Maleic Anhydride

**Scheme 30:**
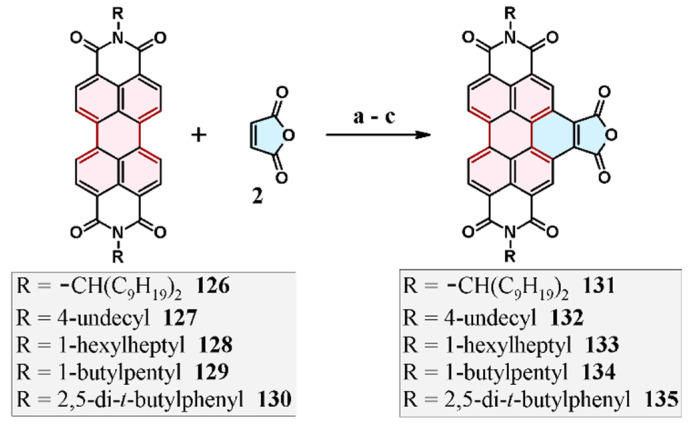
Cycloaddition of maleic anhydride to perylene bisimide [187,188,189]. Reagents and conditions: (a) for R = -CH(C_9_H_19_)_2_: *p*-chloranil, 202 °C, 12 h (at the beginning, a few milliliters of acetone were added, but the high reaction temperature indicated that it was gradually evaporating), Y = 65%; (b) for R = 4-undecyl: excess of melted *p*-chloranil, rfx, 2 d, Y = not reported; and (c) excess of dienophile, a small amount of acetone, 125 °C (at the beginning), and then rfx, 4 d, Y = 71% (**133**); Y = 77% (**134**); and Y = 67% (**135**).

The cycloaddition of melted maleic anhydride (**2**) to the bay regions of *N*-substituted perylene bisimides (**126**-**130**) could be performed in the presence of *p*-chloranil, and it resulted in monoadducts **131**-**135** (diadducts were not obtained), as illustrated in Scheme 30 [187].

An analogous reaction was described by Kelber et al. using perylene bisimide with a 4-undecyl substituent; however, the cycloadduct was not isolated: it was converted into benzo[*ghi*]perylenehexacarboxylic 1,2-bis(2-ethylhexyl)ester 4,5:10,11-bis(undecen-4-yl)imide with an overall yield of 41% [186]. Langhals and colleagues also reported the cycloaddition of this dienophile to *N*,*N*’-disubstituted perylene bisimides, which is explained in the description under Scheme 30 [188,189]. The cycloaddition of maleic anhydride (**2**) to a diazobenzoperylene bisimide derivative is also possible, and it is described in Section 7.3.

### 7.2. Cycloaddition of N-Phenyl Maleimide

**Scheme 31:**
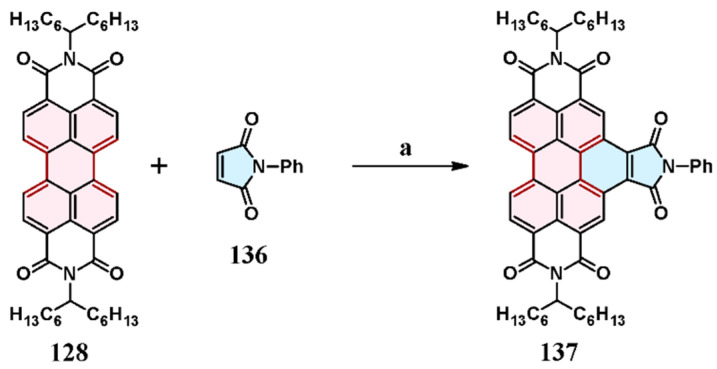
Cycloaddition of *N*-phenyl-maleimide to *N*,*N*’-bis(1-hexylheptyl)perylene bisimide [188]. Reagents and conditions: (a) = *p*-chloranil, CHCl_3_ (small amount), 120 °C, and then rfx, 3 d, Y = 94%.

Similarly, Langhals et al. presented a method for the cycloaddition reaction of *N*-phenyl-maleimide (**136**) to *N*,*N*’-bis(1-hexylheptyl)perylene bisimide (**128**), resulting in (**137**), in their U.S. patent (Scheme 31) [188].

### 7.3. Cycloaddition of 1,2,4-Triazol-3,5-Diones to Perylene Bisimides

**Scheme 32:**
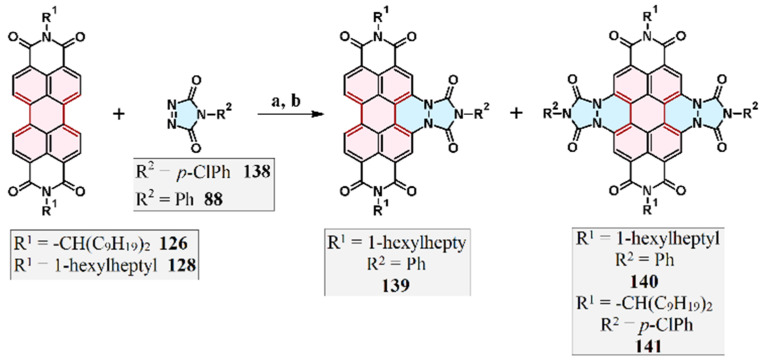
Cycloaddition of *N*-aryl-1,2,4-triazole-3,5-dione to *N*-substituted perylene bisimide [187,188,189]. Reagents and conditions: (a) for the cycloaddition of **138** to **126**: *p*-chloranil, benzene, 75 °C, N_2_, 7 d; Y = 85% of **141** (the monoadduct was not isolated) and (b) for the cycloaddition of **88** to **128**: *p*-chloranil, dry toluene, rfx, N_2_; Y = up to 41% of **139** (for **140**, yield was not reported).

In contrast to the cycloaddition reaction of maleic anhydride (to perylene bisimides), in which only the monoadduct is formed, the reaction of more reactive dienophiles, such as 1,2,4-triazole-3,5-dione derivatives (e.g., **138** and **88**), leads to both mono- (**139**) and diadduct (**140** and **141**) formation (Scheme 32) [187,188,189].

Importantly, the application of *p*-chloranil is necessary; without this oxidizing agent, the yield of cycloaddition, leading to the tetrazozacoronene (Scheme 32), was only 5% [189].

**Scheme 33:**
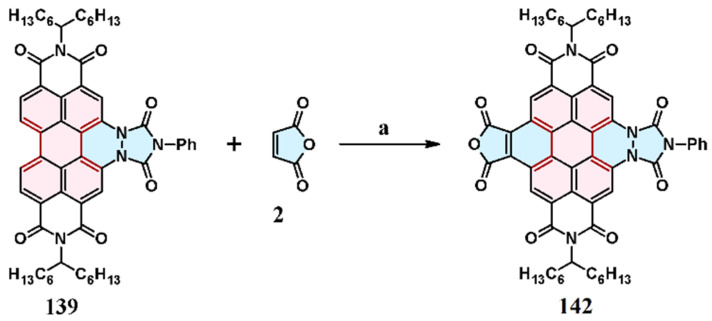
Cycloaddition of maleic anhydride to *N*,*N*′-bis(1-hexylheptyl)-2-phenyl-benzo[4.10]anthra[1,9,8-*cdef*][1,2,4]triazolyl[1,2-*a*]cinnoline-1,3-dione-5,6:11,12-bis(dicarboximide) [188]. Reagents and conditions: (a) *p*-chloranil, CHCl_3_ (small amount), 125 °C, 4 weeks; Y = 57%.

In the above-mentioned U.S. patent, the cycloaddition of maleic anhydride **2** to perylene bisimide, namely, *N*,*N*′-bis(1-hexylheptyl)-2-phenyl-benzo[4.10]anthra[1,9,8-*cdef*][1,2,4]tria-zolyl[1,2-*a*]cinnoline-1,3-dione-5,6:11,12-bis(dicarboximide) (**139**), was outlined (see Scheme 32). As a result of this reaction, a diazocoronone derivative, i.e., 11,12-diazo-11,12-dihydrocoronene-2,3,5,6,8,9,11,12-octacarboxylic acid 5,6-anhydride-2,3:8,9-bis(1-hexylheptylimide)-11,12-phenylimide (**142**), was formed, which is depicted in Scheme 33 [188].

### 7.4. Cycloaddition of Azodicarboxylate

**Scheme 34:**
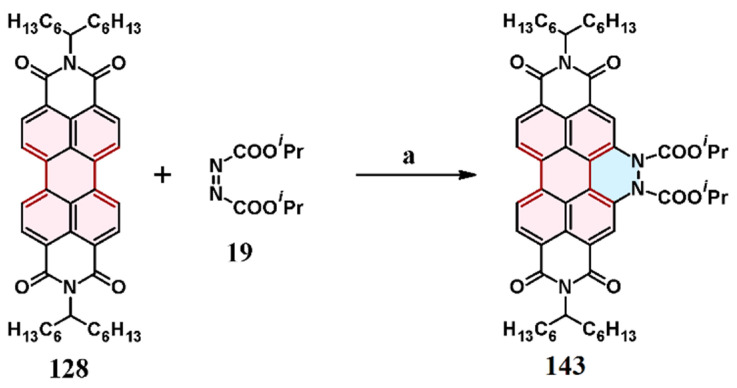
Cycloaddition of diisopropyl azodicarboxylate to *N*,*N*’-bis(1-hexylheptyl)perylene bisimide [188]. Reagents and conditions: (a) *p*-chloranil, PhMe, 140 °C, 7 d, Y = not reported.

Another reaction that appeared in Langhals et al.’s patent is the cycloaddition of diisopropyl azodicarboxylate to *N*,*N*’-bis(1-hexylheptyl)perylene bisimide (Scheme 34) [188].

### 7.5. Cycloaddition of Diarylacetylenes

**Scheme 35:**
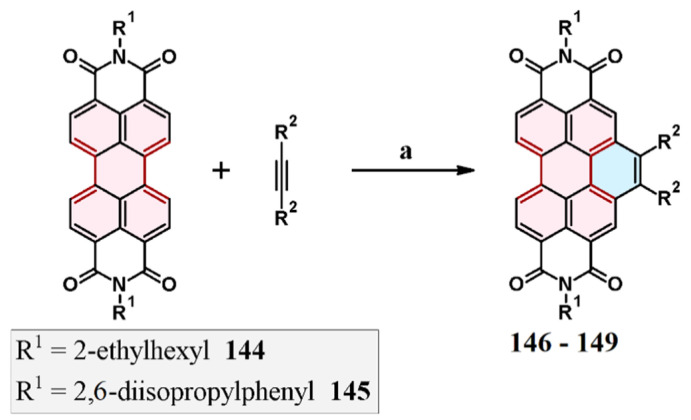
Cycloaddition of 1,2-diarylacetylenes to *N*,*N*’-bis(2-ethylhexyl- and (2,6-diisopropylphenyl)perylene bisimides bay regions [152,190,191,192,193]. Reagents and conditions: (a) 280–290 °C, 24–96 h, 0.1 Pa, Y = up to 46%, R^2^—see Figure 3.

**Figure 3:**
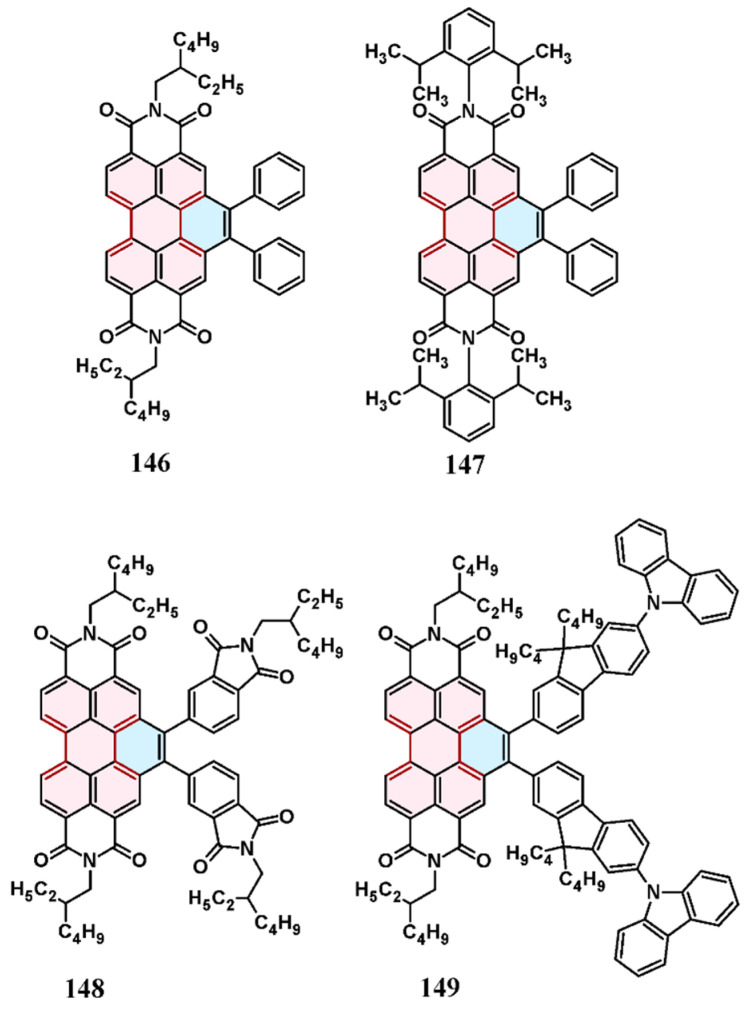
Synthesis of 2,3-diaryl-*N*,*N*’-bis(2,6-diisopropylphenyl or 2-ethylhexyl)benzo[*ghi*]perylenediimides via the cycloaddition of 1,2-diarylacetylenes to *N*,*N*’-bis(2,6-diisopropylphenyl)- or (2-ethylhexyl) benzo[*ghi*]perylene diimides bay regions: list of obtained products [152]. Y = 27% (**146**); 46% (**147**); 15% (**148**); and 10% (**149**).

Recently, we successfully performed the cycloaddition of diarylacetylenes to *N*,*N*’-disubstituted perylene bisimides (see Scheme 35 and Figure 3) [152,190,191,192,193].

The results of the reaction shown in Scheme 35 and Figure 3 were in good agreement with the results of DFT calculations (see Section 12). In particular, it turns out that, for the cycloaddition of acetylene to 1,6,7,12-tetrasubstituted perylenes, the calculated activation energy of cycloadduct formation does not specifically depend on the electronic nature of the substituents (activation energy = 35.0, 34.8, and 33.9 kcal/mol for the substituents H, NH_2_, and NO_2_, respectively) [152].

### 7.6. Cycloaddition of Arynes

**Scheme 36:**
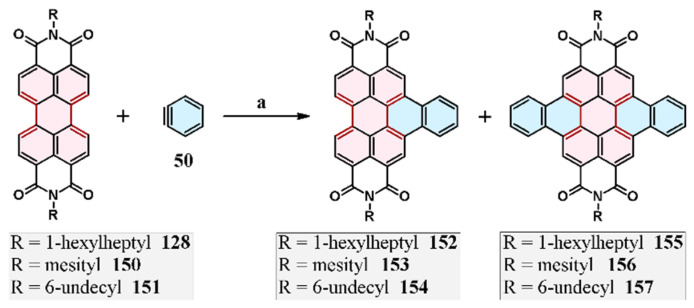
Cycloaddition of benzyne to the perylene bisimide bay region [132,133]. Reagents and conditions: (a) for **150** or **151**: benzyne precursor: 1-(OTf)-2-(TMS)benzene or 1-(COO-)-2-(N_2_+)benzene, 160 °C, 48 h; KF, PhCN [132] and (b) for **128**: 1-(OTf)-2-(TMS)benzene (70 °C, THF) or 1-(COO-)-2-(N2+)benzene (70 °C, THF, 18-Crown-6), up to 36 h [133].

In 2019, two teams almost concurrently published the results of their application of the APEX strategy (i.e., cycloaddition of arynes to perylene bisimides **128**, **150**, and **151**) for the synthesis of core-extended perylene bisimides (**152**-**157**) [132,133]. In Scheme 36, the results obtained by both teams for the reaction of benzyne (**50**) cycloaddition are shown.

**Figure 4:**
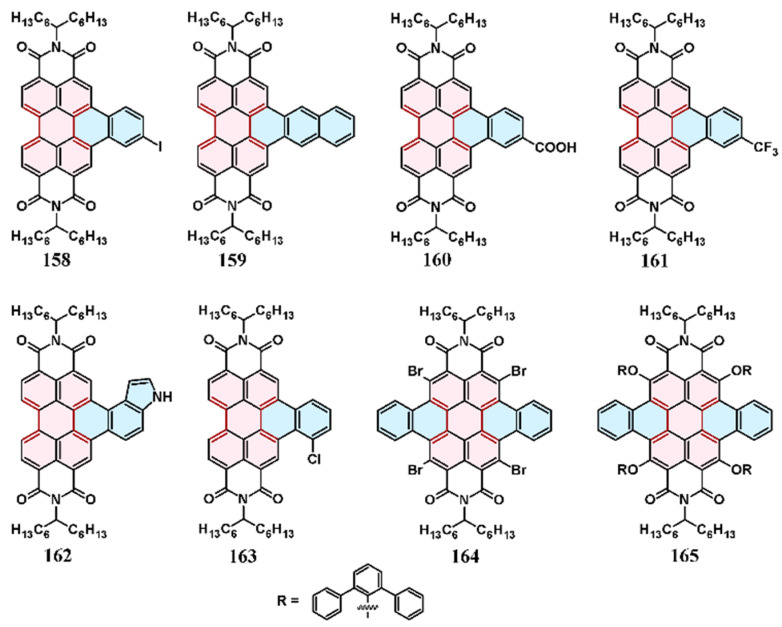
Core-expanded functionalized perylene bisimides (yields: 22–89%) [133].

Nakamura et al. obtained a mixture of mono- and dicycloaddition products, but the isolated yields were low or very low (or were not given), despite the long reaction time and high temperature [132]. The authors emphasized that they had difficulties with product isolation from the post-reaction mixtures, and as a result of these obstacles, the maximum isolated yields of the monoadduct reached only 37%. According to our experience, a high reaction temperature leads to the creation of many side products. Therefore, benzyne cycloaddition should be conducted at the lowest possible reaction temperature, preferably lower than 80 °C [178,179,180,181]. The results of the second scientific team, namely, Zink-Lore et al. [133] confirmed the role of the reaction temperature in the creation of side products. The isolated yields were higher (up to 70% mono- and 10% diadduct), even though the reaction temperature was only 70 °C. Moreover, when a shortage of benzyne was used, the monoadduct was selectively prepared. Furthermore, better results were obtained when 1-(OTf)-2-(TMS) benzene played the role of the benzyne precursor instead of 1-(COO^-^)-2-(N_2_^+^) benzene [133]. Using various aryne precursors and appropriate reaction conditions, the mentioned authors obtained several new core-extended perylene bisimides. A few of them are presented in Figure 4 [133].

**Scheme 37:**
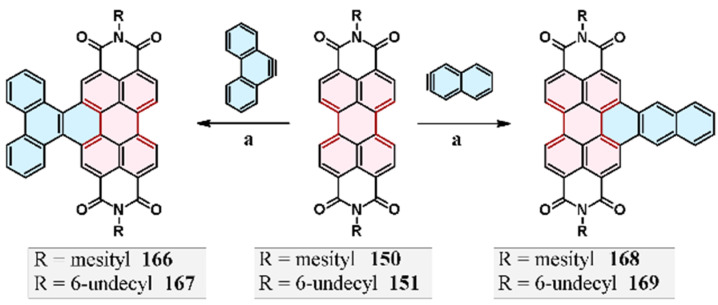
Cycloaddition of 2,3-naphthyne and 9,10-anthryne to the perylene bisimide bay region [132]. Reagents and conditions: (a) 160 °C, 48 h, PhCN, KF, Y = 54% (**166**); 54% (**167**); trace (**168**); and 36% (**169**).

The first mentioned scientific team, Nakamura and coworkers, successfully realized the cycloaddition of 2,3-naphthyne and 9,10-anthryne (generated from both corresponding precursors, namely, OTf- and TMS- or N_2_^+^- and COO^-^-substituted 2,3-naphthalene or 9,10-phenanthrene derivatives) to the bay regions of perylene bisimides (**150** and **151**), as shown in Scheme 37 [132]. The reaction conditions were identical to those shown in Scheme 36, and isolated yields reached a maximum of 54%.

The research of both above-mentioned scientific teams was supported by DFT calculations (see Section 12).

## 8. Cycloaddition to Perylenetetracarboxylic Acid Tetraesters

**Scheme 38:**
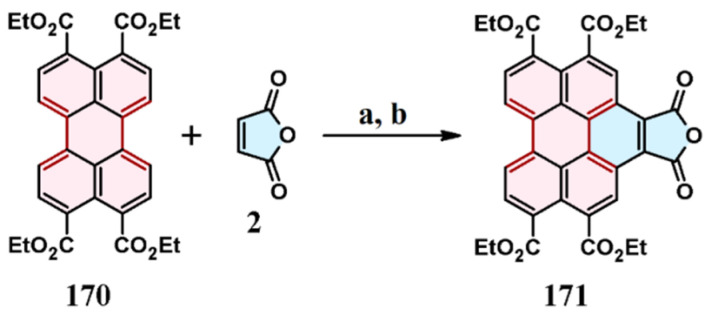
Cycloaddition of maleic anhydride to perylene 3,4,9,10-tetracarboxylic tetraethyl ester [117,186]. Reagents and conditions: (a) *p*-chloranil, rfx, 24 h, Y = not reported [186] and (b) 170 °C, 36 h, Y = 70% [117].

In 2011, Kelber et al. described the cycloaddition of maleic anhydride (**2**) to perylene 3,4,9,10-tetracarboxylic acid tetraethyl ester (**170**), as shown in Scheme 38. The cycloaddition product (**171**) was not isolated, and the authors performed a further reaction, i.e., the conversion of this cycloadduct into benzo[*ghi*]perylene-1,2,4,5,10,11-hexacarboxylic hexaethyl ester (with 53% overall yield) [186]. It is worth mentioning that the product of the cycloaddition to both bay regions was not obtained. A similar reaction was also realized in 2017 but with some modifications in reaction conditions (170 °C, 36 h, excess of melted maleic anhydride as a solvent, *p*-chloranil), which resulted in a higher, 70% yield of benzo[*ghi*]perylene 1,2-anhydride-4,5,10,11-tetracarboxylic tetraethyl ester [117].

**Scheme 39:**
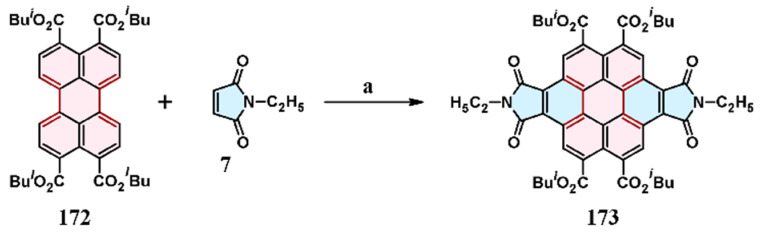
Cycloaddition of *N*-ethyl maleimide to perylene-3,4,9,10-tetracarboxylic acid tetra-iso-butyl ester: synthesis of coronene derivative [141]. Reagents and conditions: (a) *p*-chloranil, *p*-hydroxyanisole, rfx (oil bath at 240 °C), 18 h, Y = 2 to 10% (depending on the extent of saponification).

In turn, the cycloaddition of *N*-ethyl maleimide (**7**) to a tetraester, i.e., perylene-3,4,9,10-tetracarboxylic acid tetra-*iso*-butyl ester (**172**), takes place in both bay regions of the perylene core (Scheme 39) [141].

**Scheme 40:**
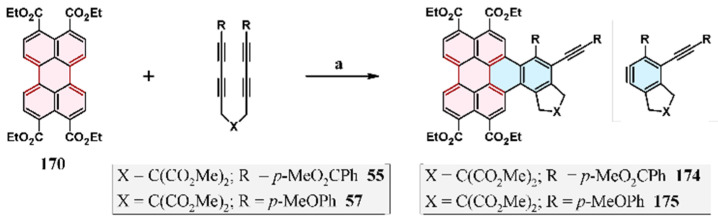
Synthesis of naphthoperylene derivatives via HDDA cascade reactions [175]. Reagents and conditions: (a) CHCl_3_, 85 °C, ~12 h; Y = 31% (**175**) and 46% (**174**).

As mentioned before (see Section 2.10), in 2016, the cycloaddition of benzyne derivatives to the bay region of perylene was reported. Furthermore, cycloaddition to their tetraethoxycarbonyl derivative (**170**) was also described (Scheme 40) [175].

**Scheme 41:**
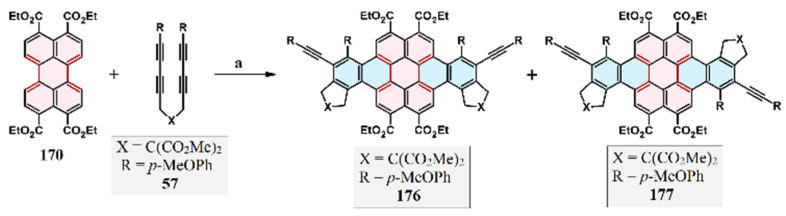
Synthesis of dibenzocoronene derivatives via HDDA cascade reactions: two isomers of the double adduct are created [175]. Reagents and conditions: (a) CHCl_3_, 85 °C, ~12 h, Y = 5% (1:2 mixture of **176**:**177**).

The formation of a small amount (5%) of the double adduct is observed in the reaction, in which **57** is involved. Furthermore, two isomers (**176** and **177**) are obtained (Scheme 41).

**Scheme 42:**
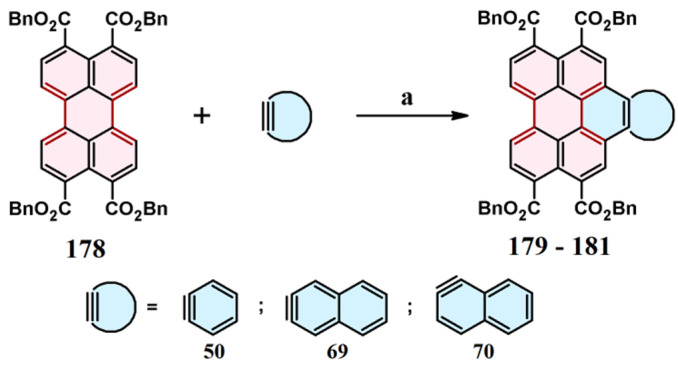
Cycloaddition of benzyne and naphthynes (1,2- and 2,3-naphthynes) to the 3,4,9,10-tetra(benzyloxycarbonyl)perylene bay region [194,195,196]. Reagents and conditions: (a) aryne precursors (2-(trimethylsilyl)phenyl trifluoromethanesulfonate (for **50**), 3-(trimethylsilyl)-2-naphthyl trifluoromethanesulfonate (for **69**), 1-(trimethylsilyl)-2-naphthyl trifluoromethanesulfonate (for **70**), CsF in 1,2-dimethoxyethane + 1,2-dicyanoethane (1*v*/1*v*), 60 °C, 24 h; Y = 75% (benzyne); and 73% (2,3-naphthyne) and 65% (1,2-naphthyne).

Recently, we developed a method for the cycloaddition of benzyne (**50**) and 2,3- and 1,2-naphthynes (**69** and **70**, respectively) to the bay region of perylene tetracarboxylic acid tetrabenzyl ester (**178**) to obtain the corresponding derivatives (**179**-**181**), as shown in Scheme 42 [194,195,196]. In this case, we also applied a novel system for aryne generation that we developed, namely, CsF + 1,2-dicyanoethene + 1,2-dimethoxyethane (also see Section 9). Reaction yields were 20–30% higher compared with the reaction using CsF in MeCN/THF solution.

Moreover, we observed that the generation of arynes using systems containing KF (in THF) was not efficient, in contrast to the reaction with the unsubstituted perylene core (see Section 2.10). It turns out that the destruction of the tetraester takes place (probably because of the competitive nucleophilic attack of fluoride on the esters groups).

## 9. Cycloaddition to Benzo[*ghi*]perylene and Its Derivatives Bay Region

**Scheme 43:**
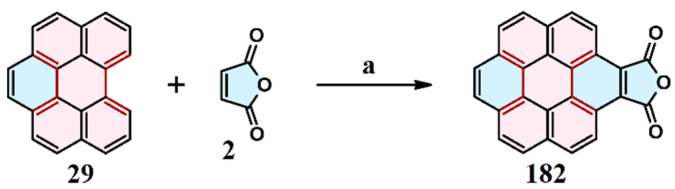
Synthesis of coronene-1:2-dicarboxylic anhydride via the cycloaddition of maleic anhydride to the benzo[*ghi*]perylene bay region [136]. Reagents and conditions: (a) *p*-chloranil (excess), rfx, 5 h; Y = 63%.

In 1957, Clar and Zander described the cycloaddition of maleic anhydride (**2**), in the presence of *p*-chloranil as an oxidant, to benzo[*ghi*]perylene (**29**), which resulted in (**182**), as shown in Scheme 43 [136]. Importantly, this reaction does not take place in nitrobenzene (used as a solvent and oxidizing agent) [135].

**Scheme 44:**
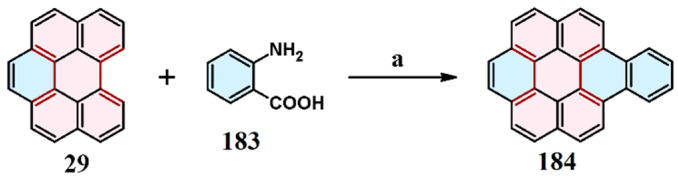
Synthesis of benzocoronone via the cycloaddition of benzyne to the benzo[*ghi*]perylene bay region [167,168]. Reagents and conditions: (a) isoamyl nitrite, DCM + THF, rfx, 8 h; Y = not reported.

Coronene derivatives could also be obtained in the reaction of bromomaleic anhydride instead of maleic anhydride, but the product of this reaction, despite purification (washing, crystallization), was contaminated with bromine. According to the authors, the above-mentioned reaction proceeded via cycloaddition with further hydrogen bromide and thermal dihydrogen elimination [136]. In the literature, the cycloaddition of maleic anhydride to benzo[*ghi*]perylene (**29**) using microwaves (MW; 850 W) is also reported [142]. Interestingly, in this reaction, benzo[*ghi*]perylene (**29**) underwent cycloaddition in nitrobenzene, while Clar [136] stated that in a similar reaction, but without the application of MW, this did not occur. In our opinion, it may be the result of MW application and significant local overheating of the reaction mixture, which has been well documented for reactions characterized by various mechanisms [136]. In a U.S. patent, the authors also described the cycloaddition of benzyne (generated from anthranilic acid (**183**) and isoamyl nitrite) to **29**, resulting in the formation of **184** (Scheme 44) [167,168].

The final product purification procedure was multistep, and chromatography on alumina was employed; however, the yield was not reported (the authors described it as “satisfactory yield”, based only on structure and purity IR analysis).

**Scheme 45:**
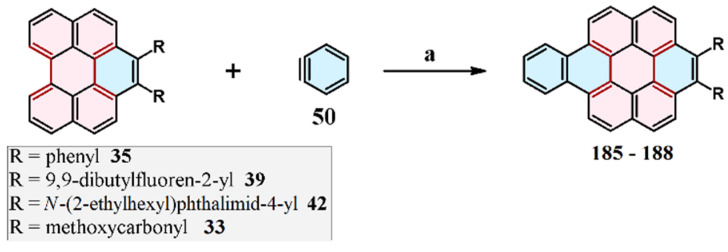
Cycloaddition of benzyne to the bay region of diaryl(or dimethoxy)benzoperylenes [152,197,198,199,200]. Reagents and conditions: (a) CsF + 1,2-dicyanoethene + 1,2-dimethoxyethane (1*v*/1*v*), 80 °C, 24 h, Y = up to 78%, R—see Figure 5.

**Figure 5:**
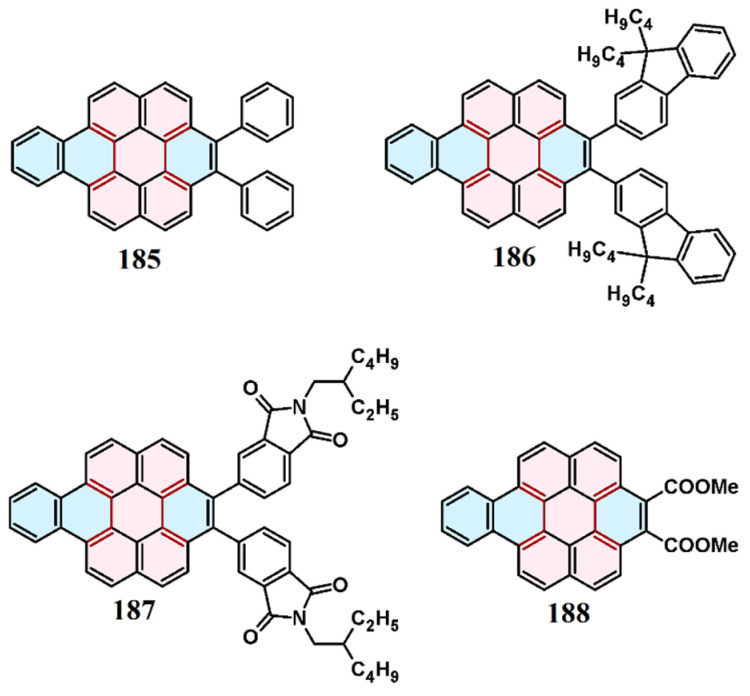
Synthesis of benzocoronenes via the cycloaddition of benzyne to selected 1,2-diarylbenzo[*ghi*]perylenes. Y = 45% (**185**); 65% (**186**); 78% (**187**); and 65% (**188**).

This year, we found that with the application of an extremely effective method of benzyne generation, cycloaddition to the second bay area of diaryl (or dimethoxycarbonyl) benzoperylenes is possible (Scheme 45 and Figure 5). In this way, coronene derivatives can be synthesized in two stages: cycloaddition of diarylacetylenes (or dimethyl acetylenedicarboxylate) to the perylene bay region (see Section 2.7 and Section 2.8, respectively) with the subsequent cycloaddition of in-situ-generated benzyne (**50**) to diaryl **35**, **39**, and **42** (or dimethoxycarbonyl **33**) benzoperylenes. This method is fully innovative and is currently the subject of patent applications [197,198,199,200]. Importantly, the CsF + MeCN + THF system is not used to obtain the products (**185**-**188**).

**Scheme 46:**
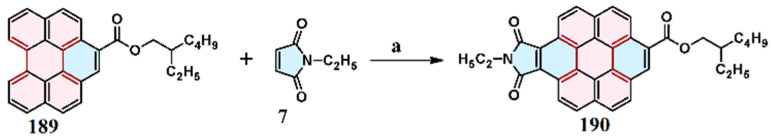
Cycloaddition of *N*-ethylmaleimide to 1-alkoxycarbonylbenzo [*ghi*]perylene [141]. Reagents and conditions: (a) *p*-chloranil, *p*-hydroxyanisole, rfx (oil bath at 240 °C), 8 h, Y = 70%.

One example of coronene derivative synthesis is the cycloaddition reaction of *N*-ethylmaleimide **7** to benzo[*ghi*]perylene-1-carboxylic (2-ethylhexyl)ester (**189**), in which coronene 1,2,7-tricarboxylic 1:2-ethylimide-7-(2-ethylhexyl)ester (**190**) is obtained (see Scheme 46) [141].

**Scheme 47:**
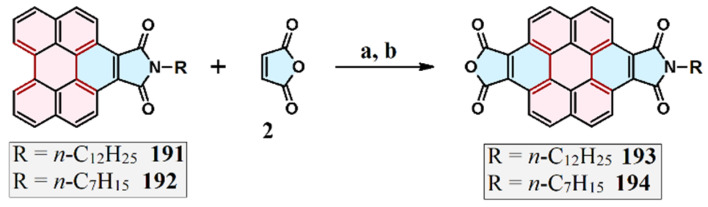
Cycloaddition of maleic anhydride to derivatives of benzo[*ghi*]perylene [140,141]. Reagents and conditions: (a) for **193**: *p*-chloranil, rfx, 4 d, Y = 48%; (b) for **194**: *p*-chloranil, rfx (oil bath at 240 °C), 4 d, Y = not reported.

Coronene derivatives (for example, **193** and **194**) can also be obtained in the reaction of maleic anhydride **2** with derivatives of benzo[*ghi*]perylenes, e.g., **191** and **192** (which are formed during the reaction of anhydride with the corresponding amine, not in the DA cycloaddition), which is shown in Scheme 47 [140,141].

**Scheme 48:**
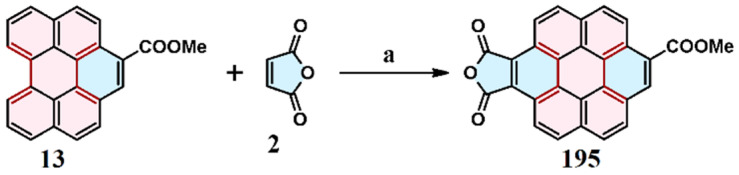
Cycloaddition of maleic anhydride to the 1-methoxycarbonyl benzo[*ghi*]perylene bay region [144]. Reagents and conditions: (a) *p*-chloranil, 220 °C, 4 h, Y = 66% (reaction yield refers to the methyl triester into which the cycloadduct was transformed).

The overall yield of **194** was not reported: it immediately underwent a transformation into *N*,*N*’-dialkyl diimide (in the reaction with heptylamine), which resulted in 66% yield, so it can be presumed that the DA reaction yield was at least that value. A coronene derivative was also obtained via the cycloaddition of maleic anhydride (**2**) to 1-(methoxycarbonyl)benzo[*ghi*]perylene (**13**), but the yield of reaction product **195** was very low (Scheme 48) [144].

**Scheme 49:**
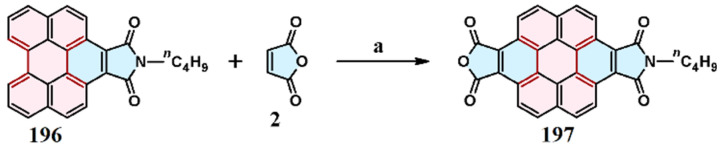
Cycloaddition of maleic anhydride to a benzo[*ghi*]perylene derivative [144]. Reagents and conditions: (a) *p*-chloranil, 220 °C, 3 d; the product was transformed into a tetraester without isolation.

In the same work, the cycloaddition of **2** to *N*-(*n*-butyl)benzo[*ghi*]perylene-1,2-dicarboximide **196**, leading to the creation of coronene derivative **197** (not isolated), was reported (Scheme 49) [144]. The final product (after hydrolysis to tetracarboxylic acid and then methylation) was coronene-1,2,7,8-tetracarboxylic acid tetramethyl ester, which was obtained with 38% yield [144].

**Scheme 50:**
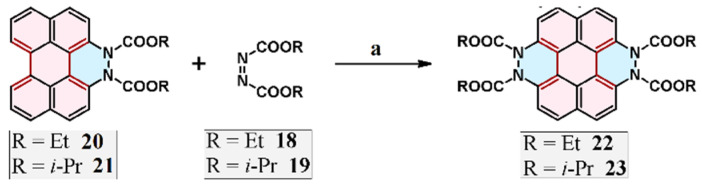
Synthesis of tetraazocoronene derivatives via the cycloaddition of dialkyl diazene dicarboxylates to diazoperylene (or perylene) bay region [145]. Reagents and conditions: (a) no solvent, 160 °C, 6 h, Y = 56% (**22**) and (b) no solvent 160 °C, 12 h, Y = 28% (**23**).

As previously stated, Tokita et al. described the cycloaddition of dialkyl diazenedicarboxylates **18** and **19** to perylene (**1**) (see Section 2.4), which resulted in **20** and **21**, respectively, and to dibenzo[*b,n*]perylene (**199**) (Scheme 52) [145]. They also performed a similar cycloaddition to the bay regions of 1,2-diazobenzo[*ghi*]perylenes **20** and **21**, and tetraazocoronene derivatives **22** and **23** were obtained (Scheme 50) [145].

Interestingly, when bare perylene was used as a substrate instead of diazobenzoperylenes, the isolated yield of tetrazocoronene was lower (see Scheme 8).

**Scheme 51:**
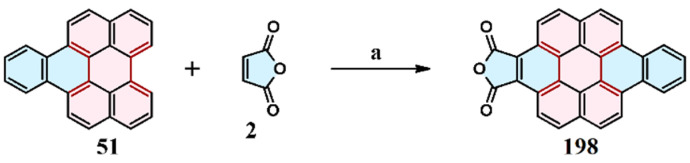
Cycloaddition of maleic anhydride to the naphtho[*ghi*]perylene bay region [167]. Reagents and conditions: (a) *p*-chloranil, rfx, 1 h, Y = 70%.

Matsuda and colleagues presented the cycloaddition of maleic anhydride (**2**) to naphtho[*ghi*]perylene (**51**) to afford the desired product (**198**) in 70% yield (Scheme 51) [167]. However, they confirmed the structure of the obtained coronene derivative using only the IR method.

**Scheme 52:**
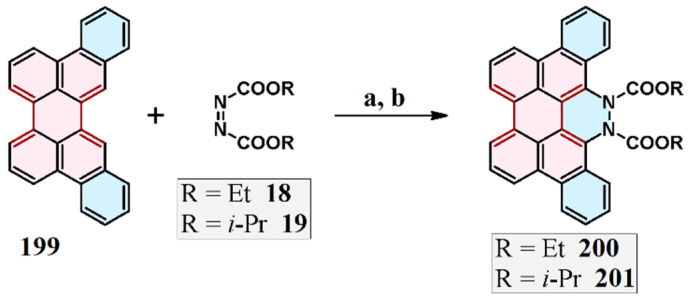
Synthesis of 1,2-diazobenzo[*ghi*]perylene via the cycloaddition of dialkyl diazene dicarboxylates to the perylene bay region [145]. Reagents and conditions: (a) for **200**: benzene, rfx, 5 h, Y = 87% and (b) for **201**: no solvent, 160 °C, 2 h, Y = 73%.

Furthermore, Tokita et al. also carried out the cycloaddition reaction of dialkyl diazenedicarboxylates **18** and **19** to the bay region of dibenzo[*b,n*]perylene (**199**), and as a result, corresponding monoadducts **200** and **201** were obtained (Scheme 52) [145].

On the other hand, benzo[*ghi*]perylene (**29**) does not react with dialkyl diazodicarboxylates (i.e., **18** and **19**) under the same conditions, which correlates well with the lack of reactivity of the latter toward maleic anhydride (**2**) [34].

## 10. Cycloaddition to Tribenzoperylene

**Scheme 53:**
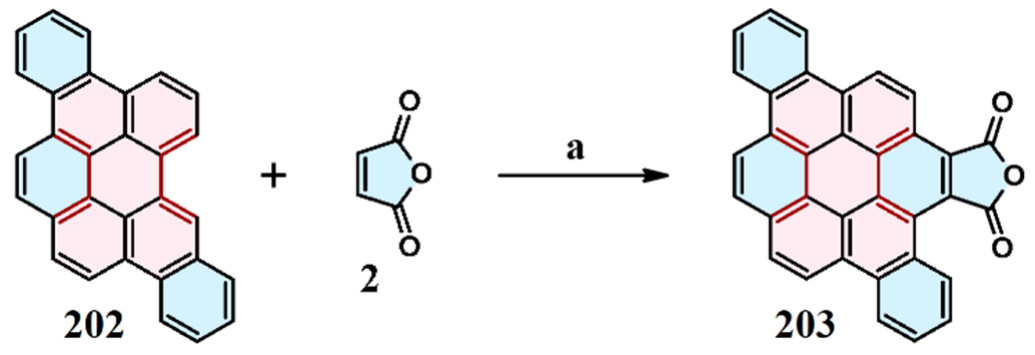
Synthesis of 1:2-7:8-dibenzocoronene-3:4-dicarboxylic anhydride via cycloaddition–rearomatization of maleic anhydride to the 1:12-2:3-8:9-tribenzoperylene bay region [136]. Reagents and conditions: (a) *p*-chloranil, rfx, 1.5 h, Y = 88%.

In their work, Clar and Zander also presented the cycloaddition of maleic anhydride (**2**) to 1:12-2:3-8:9-tribenzoperylene (**202**), producing 1:2-7:8-dibenzocoronene-3:4-dicarboxylic anhydride (**203**), as shown in Scheme 53 [136].

The same product (**203**) may also be formed in the reaction in which bromomaleic anhydride is used instead of maleic anhydride (no solvent, rfx 2 h, Y = 60%). This reaction is believed to proceed according to the following mechanism: typical cycloaddition and then thermal rearomatization via HBr and H_2_ elimination. In 1969, Zander published a work regarding the relative reactivity of perylene and other polycyclic aromatic hydrocarbons based on a perylene core in a cycloaddition reaction of maleic anhydride to the bay region (see Section 12).

## 11. Cycloaddition to Bisanthene and Its Derivatives

### 11.1. Cycloaddition of Maleic Anhydride

**Scheme 54:**
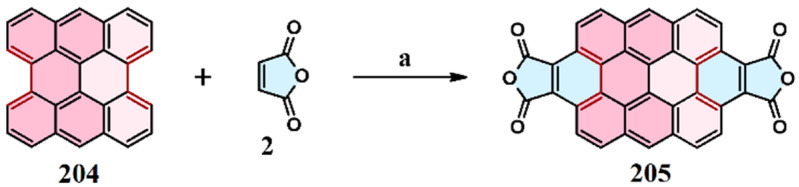
Cycloaddition of maleic anhydride to both bisanthene bay regions [202,203]. Reagents and conditions: (a) nitrobenzene (solvent and oxidant), rfx, Y = quant.

**Scheme 55:**
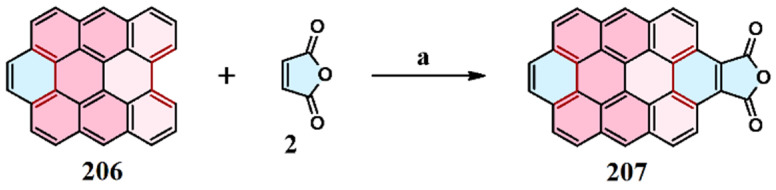
Cycloaddition of maleic anhydride to the benzobisanthene bay region [202,203]. Reagents and conditions: (a) nitrobenzene (solvent and oxidant), rfx, Y = quant.

In 1948, Clar published a paper about the cycloaddition of maleic anhydride (**2**) to the bay regions of bisanthene (**204**) and benzobisanthene (**206**) to obtain **205** and **207**, respectively (see Scheme 54 and Scheme 55) [201,202]. This publication was also cited in a review in 2015 [203].

Bisanthene (**204**) turned out to be significantly more reactive than bare perylene (**1**); therefore, the cycloaddition takes place in both bay regions and occurs, interestingly, quantitatively. Further calculations for the cycloaddition of acetylene and other dienophiles (see Section 12) at DFT levels confirmed that the activation energy of the cycloaddition reaction to the **204** bay region is much lower than that for **1**. Both of the above-mentioned reactions were applied in the synthesis of ovalene via cycloaddition–decarboxylation [202,203].

### 11.2. Cycloaddition of Acetylene

**Scheme 56:**
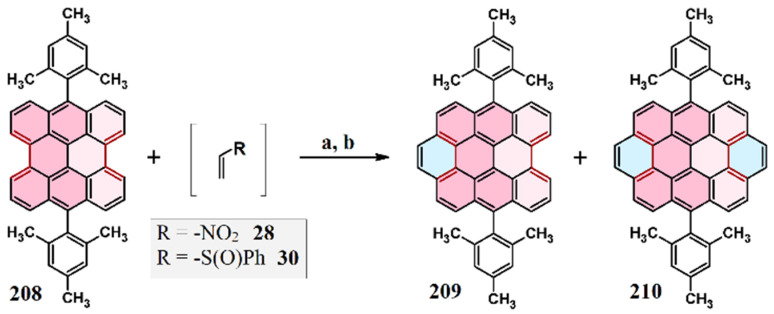
Mono- and double-cycloaddition of acetylene (nitroethene and vinyl sulfoxide as an acetylene source, “masked acetylene”) to the bisanthene bay region [147]. Reagents and conditions: (a) 2-nitroethanol (“masked” acetylene source), phthalic anhydride, pressure vessel, 135 °C, PhMe, 1 d, Y = 84%; (b) phenyl vinyl sulfoxide (“masked” acetylene source), 155 °C, no solvent, 1 d, Y = 16.5%

In 2010, the synthesis of 7,14-dimesitylovalene (**210**) via the double-cycloaddition of acetylene, generated in situ from nitroethene (**28**) or phenyl vinyl sulfoxide (**30**) as masked acetylene, to 7,14-dimesitylbisanthene (**208**) was reported by Fort and Scott (Scheme 56) [147].

The final product (**210**) of double-cycloaddition was obtained with 84% (nitroethene) or 16.5% (phenyl vinyl sulfoxide) yield. When pure nitroethylene was applied as the acetylene source, a mixture of mono- and diadducts (**209** and **210**, respectively) was formed. As mentioned in Section 2.6, it is a two-step process: the first step is the cycloaddition of nitroethene **28**, followed by the thermal elimination of HONO. Interestingly, the application of pure nitroethene did not yield better results. Generally, bisanthene (**204**) is a much more reactive diene than perylene (**1**) in the DA cycloaddition to the bay region, which is shown both experimentally and in computational studies (also see Section 12) [147]. It should be added that the mesityl group is very important not only because of the solubility and π-stacking limitation but primarily because of the electronic structure of this polyaromatic system. Bisanthene (**204**) can also exist as an open-shell PAH in the form of a diradical [1]. The introduction of any bulky group, such as mesityl, into the 7- and 14-positions of bisanthene is one of the approaches to reducing the reactivity of bisanthene as biradicals [1].

**Scheme 57:**
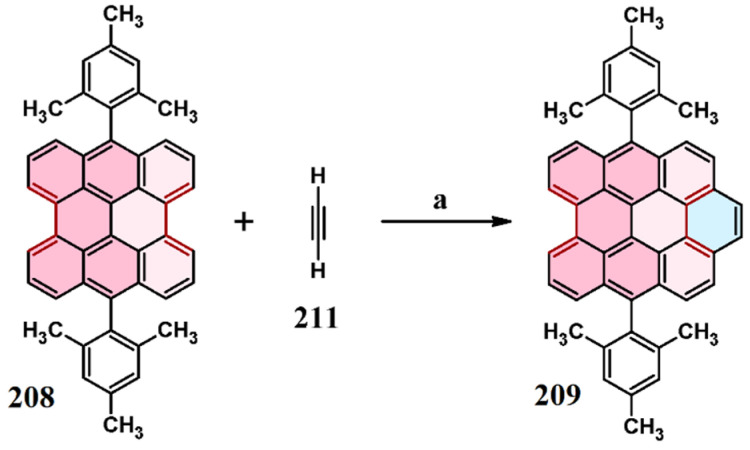
Cycloaddition of acetylene to 7,14-di(mesityl)bisanthene [204]. Reagents and conditions: (a) 1.8 atm, DMF, 140 °C, 48 h (substrate:product = 21:79) or 7 d (substrate:product = 34:66).

In 2012, the cycloaddition of acetylene gas (**211**), dissolved in DMF, to 7,14-disubstituted bisanthene was described, and the monoadduct (**209**) was created (Scheme 57) [204]. Prolongation of the reaction time from 48 h to 7 days caused the molar ratio of product:substrate to increase from 21:79 to 34:66, but, unfortunately, undesired side reactions were observed. In turn, when toluene or 1,2-dichlorobenzene was used as a solvent, conversion of this reaction was lower than that in DMF (the solubility of acetylene gas in DMF is exceptionally good).

Due to the explosive properties of acetylene (**211**), the procedure requires strict pressure control in the reactor [204].

### 11.3. Cycloaddition of Acetylenedicarboxylate to Di-Mesityl-Bisanthene

**Scheme 58:**
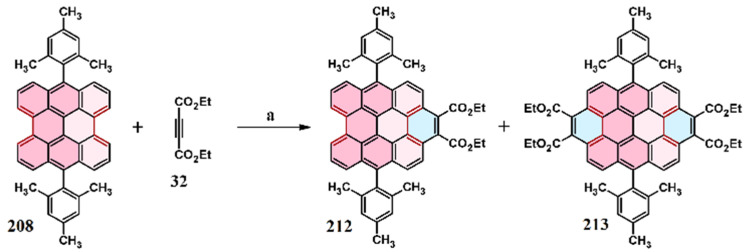
Cycloaddition of diethyl acetylenedicarboxylate to the bisanthene derivative [149]. Reagents and conditions: (a) excess of dienophile, toluene, 120 °C, Y = 44% (**212**) and 12% (**213**).

The cycloaddition of acetyledicarboxylates to the bisanthene (**204**) bay region is much easier than in the case when perylene (**1**) is applied as a diene, and this was confirmed experimentally using both dienes (no-perylene adduct was observed) [148]. In contrast, in the reaction of bisanthene derivative **208** with diethyl acetylene dicarboxylate (**32**), 100% conversion into diadduct **213** is possible (Scheme 58) [149]. Our recent research indicates that for perylene, even at a temperature of 185 °C, no diadduct is created, but 100% conversion of perylene to the monoadduct is achieved (see Section 2.7).

When the reactions shown in Scheme 58 proceeded at 120 °C for 24 h using 50 eq. of diethyl acetylenedicarboxylate (**32**), the conversion was 100%, and only diadduct **213** was detected in the post-reaction mixture. Decreasing the dienophile amount resulted in the formation of mono- and diadducts (**212** and **213**, respectively), or even substrate **208** and monoadduct **212**. The results of the experiment were supplemented by DFT calculations (see Section 12).

### 11.4. Cycloaddition of Arynes

**Scheme 59:**
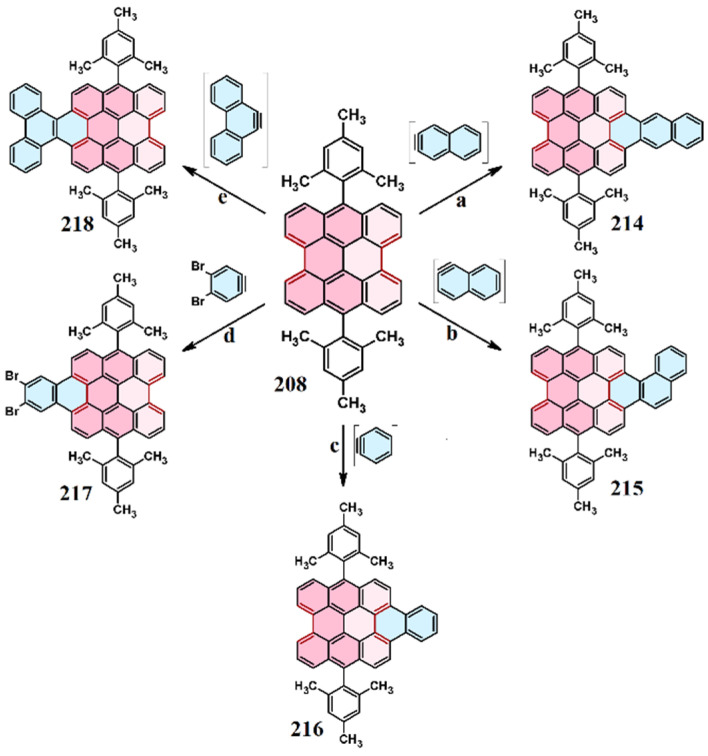
Cycloaddition of in situ generated arynes to the 7,14-di-mesityl-bisanthene, i.e., 7,14-dimesitylphenanthro[1,10,9,8-*opqra*]perylene [205]. Reagents and conditions: (a) 3-amino-2-naphthoic acid, isoamyl nitrite, PhMe, rfx, 1 h, Y = 39%; (b) 1-bromonaphthalene, NaNH_2_, THF, rfx, 2 h, Y = 49%;(c) 2-(trimethylsilyl)phenyl trifluoromethanesulfonate, CsF, THF, rfx, 1 d, Y = 72% or anthranilic acid, isopentyl nitrite, PhMe, 1 h, Y = 75%; (d) *n*-BuLi, 1,2,4,5-tetrabromobenzene, PhMe, rfx, 1.5 h, Y = 51%; and (e) 9-bromophenanthrene, NaNH_2_, THF, rfx, 12 h, Y = 40%.

In 2013, cycloadditions of various in-situ-generated arynes to 7,14-di-mesityl-bisanthene, i.e., 7,14-dimesitylphenanthro[1,10,9,8-*opqra*]perylene (**208**), were described (Scheme 59) [205]. As a result of these reactions, a number of products (**214**-**218**) were synthesized.

All reactions proceeded smoothly under mild conditions, and it should be noted that the monoadduct could be obtained as the dominant product [205].

### 11.5. Cycloaddition of Benzo- and Naphthoquinones

**Scheme 60:**
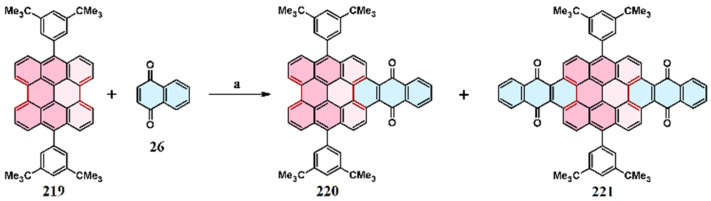
Cycloaddition of 1,4-naphthoquinone to the bisanthene derivative bay region [206]. Reagents and conditions: (a) PhNO_2_, (oil bath at 240 °C), rfx, 1 day (**220** was the major product, and only a trace amount of **221** was formed) or 2 days (**221** was the major product, and only a trace amount of **220** remained).

The cycloaddition of 1,4-naphthoquinone (**26**) to bisanthene derivative **219** was reported in 2011 (see Scheme 60) [206]. In this reaction, nitrobenzene acts both as a solvent and, primarily, an oxidizing and dehydrogenation agent. There are 3,5-di-*tert*-butylphenyl groups in the structure of the molecule that affect the stability and solubility of the substrate and products.

The yield of this reaction was not reported (the mixture of final products **220** and **221** was subjected to further transformations); however, it can be concluded that it was ca. 60% if we take into account the yield of post-conversion final products [206].

**Scheme 61:**
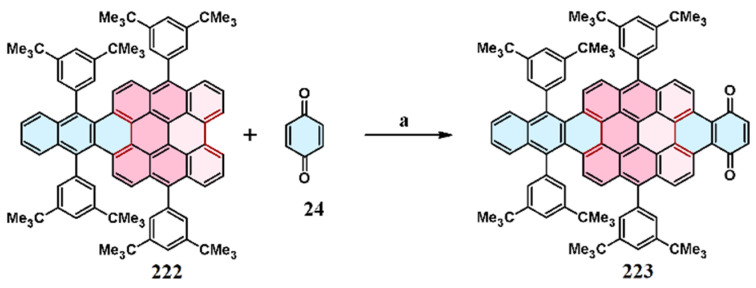
Cycloaddition of 1,4-benzoquinone to the bisanthene derivative bay region [206]. Reagents and conditions: (a) PhNO_2_, rfx, 2 d, Y = 85%.

The bisanthene derivative obtained as a product of the mono-cycloaddition of 1,4-naphthoquinone (**26**) (see Scheme 60) was used in a further reaction with 1,4-benzoquinone (**24**), which resulted in 85% isolated yield of corresponding cycloadduct **223** (Scheme 61) [156]. 3,5-Di-tert-butylphenyl motifs present in the structure were introduced into the molecule via reaction with ArMgBr [206].

**Scheme 62:**
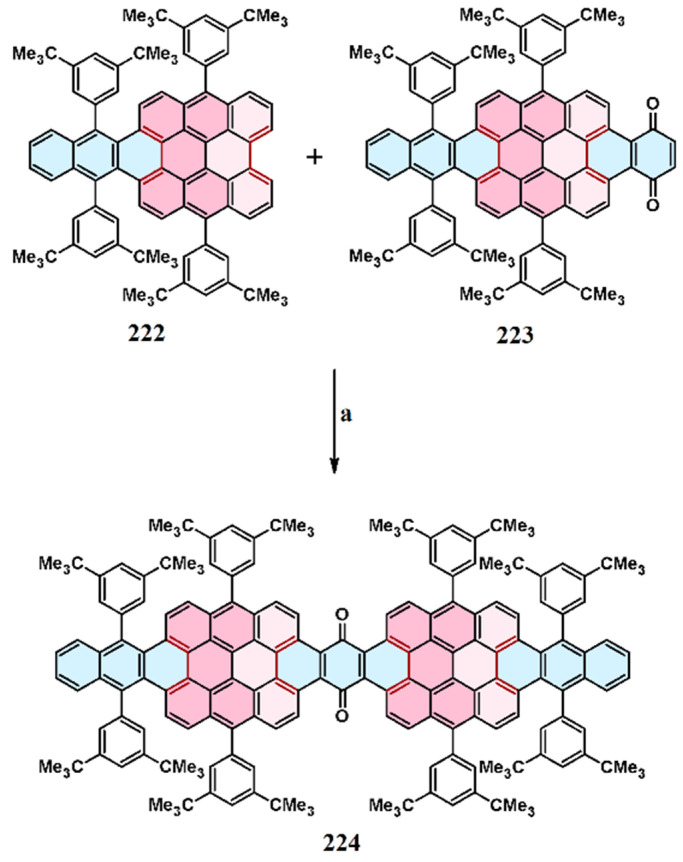
Cycloaddition to the bay region: bisanthene derivative as a diene and also as a dienophile [206]. Reagents and conditions: (a) PhNO_2_, rfx, 2 days, Y = 40%.

Cycloaddition product **223** was employed to combine with a derivative that had an unsubstituted bay region, i.e., **222** (see Scheme 60 and Scheme 61) to obtain **224**, which is shown in Scheme 62 [206].

In the above-described reaction, bisanthene motifs were connected via DA cycloaddition to the bay region: one of them was employed as a diene and the other as a dienophile [206]. Intriguingly, attempts to obtain the product from Scheme 62 in a one-step reaction (double DA) between benzoquinone and the substrate from Scheme 61 have failed [206].

**Scheme 63:**
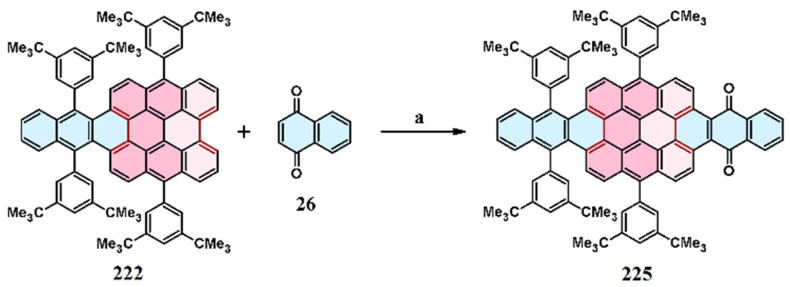
Cycloaddition of naphthoquinone to the bisanthene derivative bay region [206]. Reagents and conditions: (a) PhNO_2_, (oil bath at 240 °C), rfx, 1 d, Y = 85%.

A bisanthene derivative (substrate **222** from Scheme 61) was also used in the cycloaddition with 1,4-naphthoquinone (**26**) to produce **225**, which is shown in Scheme 63 [206].

**Scheme 64:**
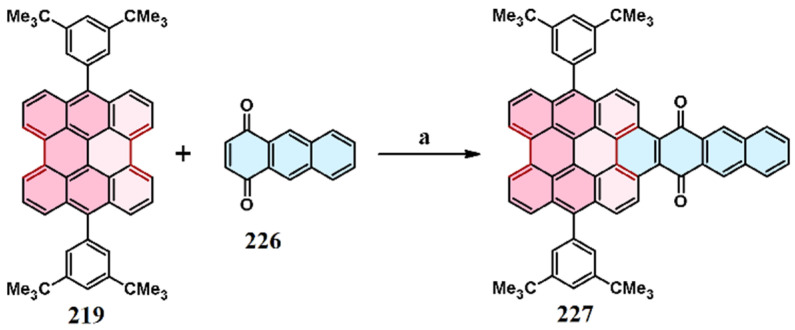
Cycloaddition of 1,4-anthraquinone to the bisanthene derivative [206]. Reagents and conditions: (a) nitrobenzene, rfx, 2 d, Y = not reported.

In the same work, the cycloaddition of 1,4-anthraquinone (**226**) to **219** was also presented (Scheme 64) [156]. As a result, cycloadduct **227** was obtained.

Monoadduct **227** (only a trace amount of the diadduct was observed) was used in further reactions. The cycloaddition reaction from Scheme 64 should result in a yield of not less than 54% after accounting for transformations that occur beforehand [206]. An extended DA time did not generate more of the diadduct, presumably because of the relatively low reactivity and low solubility of the monoadduct [206].

## 12. Computational Investigations of the Diels–Alder Cycloaddition to Perylene and Its Analogs’ Bay Regions

**Figure 6:**
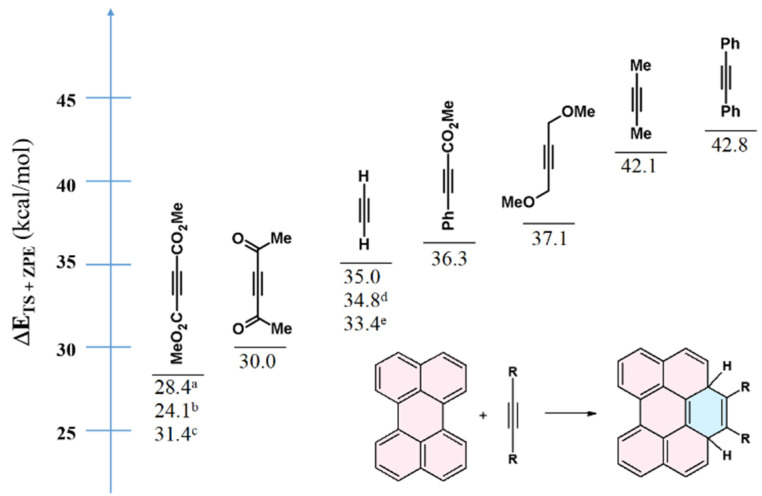
Calculated energy barriers for transition states (ΔE_TS_) in modeled cycloaddition reactions of R-CC-R to perylene and tetraamino- and tetranitro perylene bay regions, obtained by the DFT/B3LYP/def2-TZVP method [152]. Results with superscripts a, b, and c correspond to the cycloaddition of acetylenedicarboxylic acid to perylene, tetraaminoperylene, and tetranitroperylene, respectively. Superscripts d and e relate to energy barriers for the reaction of acetylene with tetraaminoperylene and tetranitroperylene.

For the first time, in 2009, the cycloaddition reaction to PAH bay regions became the object of theoretical research using the DFT method [149]. Specifically, Fort et al. hypothesized that activation energies of the cycloaddition of maleic anhydride to the bay regions of increasingly long PAHs should decrease in the sequence phenanthrene-perylene-bisanthene-tetrabenzocoronene because of the diminishing aromatic stabilization energies of the terminal “wing” benzene rings [149]. This hypothesis was supported by B3LYP/6-31G* calculations and confirmed experimentally: maleic anhydride undergoes cycloaddition to substituted bisanthene but not to perylene. The elegant approach to the benzannulation of bay areas of large polyaromatic hydrocarbons, described by Fort and Scott [147] (see Section 2.6), was discovered through the computational investigation of reactive dienophiles for this cycloaddition: nitroethene, at the B3LYP/6-31G* level of theory, was predicted to be more reactive by four orders of magnitude. In this case, the calculated activation energy for the cycloaddition of nitroethene is 8.8 kcal/mol lower relative to the energy barrier for the reaction with phenyl vinyl sulfoxide. It is worth mentioning that calculations with the use of the B3LYP functional and the 6-31G* basis set but without the dispersion corrections lead to unsystematic errors on the order of tens of kcal/mol [207]. Cao et al. performed a comprehensive study of the Diels-Alder reactivity of graphene models with 2,3-dimethoxybutadiene (DMBD), 9-methylanthracene (MeA), tetracyanoethylene (TCNE), and maleic anhydride (MA) using the M06-2X and ωB97X-D functionals [208]. None of the investigated reagents was able to functionalize interior bonds of the graphene model, but all of them are predicted to undergo facile cycloaddition to graphene edges: DMBD and MeA via [4 + 2] cycloadditions, and TCNE and MA via both [4 + 2] and [2 + 2] cycloadditions. Cycloaddition to zigzag edges (essentially, bay regions) was found to be facilitated by the higher spin density (radical character) of these regions of graphene models. According to the transition state theory, the kinetically limiting reaction step involves overcoming the energy barrier to reach the transition state (TS). For the studied cycloaddition reactions, on the basis of theoretical models of these reactions, transition state structures and activation energies were determined by applying the DFT/B3LYP level of theory and def2-TZVP basis set. Our calculations showed that, surprisingly, the activation energy of the cycloaddition reaction of disubstituted acetylenes to the bay regions of perylene and its derivatives depends mainly on the dienophile, not the diene structure [152]. A summary of these findings is presented in Figure 6.

**Figure 7:**
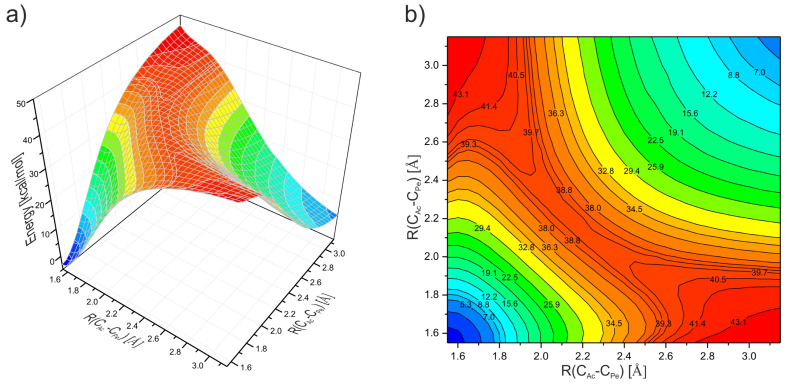
(**a**) Potential energy surface (PES) as a function of C_Ac_-C_Pe_ distances for the cycloaddition reaction of diphenylacetylene with perylene. (**b**) Vertical projection of PES in the contour representation.

The results of these calculations were crucial in planning the conditions of cycloaddition reactions of various acetylenes (R-C≡C-R type) to perylene and its derivatives. From a mechanistic point of view, the calculations showed the synchronous formation of σ bonds of the two carbon atoms of the acetylene motif in dienophiles (C_Ac_) with the respective carbon atoms of the bay regions of perylene (C_Pe_). In the first phase of the reaction, both C_Ac_ atoms attack the perylene simultaneously, and in the transition state (TS), C_Ac_⋯C_Pe_ distances for both pairs of carbon atoms are the same. In Figure 7, the calculated potential energy surface (PES) as a function of C_Ac_⋯C_Pe_ distances is presented for the cycloaddition reaction of diphenylacetylene with perylene. As is visible in Figure 7, the minimum energy path from reactants to the transition state proceeds along the diagonal of the PES, so at each moment, the formation of σ bonds between corresponding carbon atoms will proceed synchronously. For relaxation from the TS to the transition product, the same process characteristic can be observed. For the disubstituted acetylenes presented in Figure 6, in the transition state, the newly formed C_Ac_-C_Pe_ bonds have the same length if the R substituents in the dienophile are the same. If the R groups are different, i.e., PhC_2_COOMe, the calculated transition state geometry suggests that, to some extent, C-C bond formation can be an asynchronous process. For the above-mentioned dienophile, the length difference between two C_Ac_-C_Pe_ bonds is about 0.47 Å. A similar asynchronous TS is also observed for 1,2-diacetylacetylene.

In 1969, Zander published a work regarding the relative reactivity of perylene and other polycyclic aromatic hydrocarbons based on the perylene core in the cycloaddition reaction of maleic anhydride to the bay region. The results of these studies, obtained by measuring the reaction rates, are presented in Table 1 [201].

According to the author, with the exception of perylene, the sequence of relative reaction rates of the remaining dienes is ordered. Unfortunately, the reasons for the differences in the reactivity of perylene are not provided. In our opinion, such research is not possible without theoretical calculations, which were not appreciated for the analysis of these types of reactions until 2009. Our calculations for the cycloaddition of diphenylacetylene to the perylene and benzoperylene bay regions showed that the activation energy of this reaction is higher for benzoperylene (47 kcal/mol) than for perylene (43 kcal/mol) [152]. On the other hand, comparing the calculated activation energies for the reaction of acetylene with perylene and bisanthene, a significant drop (about 6 kcal/mol) in the calculated energy barrier is observed for the cycloaddition reaction with bisanthene compared with the reaction with perylene. The calculated energy barriers to the TS in the mentioned reactions amount to 35 kcal/mol and 29 kcal/mol for perylene and bisanthene, respectively [149,152].

In 2019, two research groups almost simultaneously published the results of research on the application of the APEX strategy, i.e., the cycloaddition of arynes to perylene bisimides (PBIs) for the synthesis of core-extended PBIs [132,133]. The varied reactivities of the arynes and the properties of the obtained compounds have been successfully explained using DFT calculations. T. Nakamuro et al. concluded that DFT calculations at the B3LYP/6-31G(d) level suggested that the bay-region APEX reaction between benzyne and PBI, benzo-PBI, and phenathro-PBI probably proceeds through an inverse-electron-demand Diels–Alder reaction with calculated free-energy reaction barriers of about 14.9 (PBI), 17.1 (benzo-PBI), and 15.7 (phenanthro-PBI) kcal/mol [132]. For the cycloaddition reaction of benzyne with perylene and its various derivatives, our preliminary DFT/B3LYP calculations with a def2-TZVP basis set result in very similar values of calculated free-energy reaction barriers, which are about 17–18 kcal/mol. As a general point, it should be stressed that of all theoretically studied cycloaddition reactions with different dienophiles, reactions with benzyne are characterized by the lowest energy barriers to the TS. In the work of N. Zink-Lorre et al., it was stated that theoretical calculations performed using the DFT method are able to explain the unexpected changes observed for the electronic, optical, and redox properties of the PBIs upon transversal core extension of up to three different levels [133]. According to the results of DFT calculations, transversal core extension to so-called level 1 by inserting a C=C double bond in one or two sides of the PBI system generally leads to an increase in the HOMO–LUMO energy gap relative to the unsubstituted PBI system. The HOMO-LUMO energy gap is a simple energy difference between the energies of the Highest Occupied and Lowest Unoccupied Kohn-Sham orbital obtained in the DFT calculation. In this case, the increase in the energy gap is caused by the stabilization and destabilization of HOMO and LUMO orbitals, respectively, as a result of the formation of benzene-like HOMOs and a larger number of antibonding interactions in the LUMO. These changes in the electronic structure explain, among other observations, the hypsochromic shift of the lowest electronic transition in the absorption spectrum for PBI when it is transversely extended to level 1. The π-expansion of the PBI core to level 2 by incorporating four additional carbon atoms in the form of C=C–C=C fragments, leading to asymmetric NPDI and symmetric DBCDI, also causes a hypsochromic shift in the absorption and emission band. For this expanded PBI, a reduction of the electron-accepting ability is also observed, which is apparent by lowering the value of the theoretically calculated electron affinity and using more negative reduction potentials measured experimentally. In line with DFT results, an increase in the HOMO–LUMO gap is observed as a result of the level-2 PBI core extension. DFT calculations indicate that the HOMO energies are barely affected by this π-extension to level 2, but additional peripheral benzene rings contribute to the destabilization of the antibonding interactions, leading to a significant increase in LUMO energy. As a result of connecting another C=C–C=C motif to an asymmetric NPDI or symmetric DBCDI, a level-3 transversal core extension is obtained. In contrast to level 2, the level-3 π-expansion results in the reduction of the HOMO–LUMO gap because of the increase in the π-conjugation and destabilization of the HOMO orbital. These computational results nicely correlate with a much lower measured oxidation potential and a decrease in the value of the theoretically calculated first ionization potential for PBI expanded to level 3. For derivatives with such structurally enlarged PBI cores, HOMO orbital destabilization and the reduction of the energy gap affect spectroscopic properties by decreasing the excitation energy for the lowest S_1_ singlet state, which leads to a bathochromic shift of both the lowest absorption and emission band. It is worth noting that the structural extension of the PBI core to level 2 or 3 changes the electronic structure in general by increasing the π-conjugation, and it affects the final character of the HOMO or LUMO orbitals. Observed changes in the topology of frontier orbitals lead to the reduction of the HOMO–LUMO overlap, and consequently, for the core-extended derivatives, the calculated oscillator strengths of the lowest S_0_ → S_1_ electronic excitation remain decreased. This effect successfully reproduces the experimentally observed drop in intensity for the lowest absorption band. The similar stabilization and destabilization of frontier orbitals and the influence of the energy gap change on spectral and electrochemical properties were also observed for many functionalized PAHs [152].

## 13. Importance of Cycloaddition to the Bay Regions of Perylene and Its Derivatives for Chemistry and Technology: Current Status and Perspectives

The oldest known cycloaddition to the bay region, i.e., the cycloaddition of maleic anhydride, was first developed for perylene, followed by benzo- and dibenzoperylenes and some other PAHs, including functionalized ones (perylene bisimides, dicyanoperylene, perylenetetracarboxylic acid tetraesters, benzoperylene, and their derivatives). However, the final products of these reactions, i.e., benzoperylenedicarboxylic acid anhydrides, are weakly soluble or extremely insoluble in common organic solvents, even when solubilizing (alkyl) groups are attached to the diene. It could be supposed that this is the reason that the cycloaddition of maleic anhydride to both perylene bay regions (creation of coronene) is not observed. Only in the case of bisanthene does this reaction take place in both bay regions and, moreover, does so quantitatively; this is probably because the reactivity of bisanthene is much higher than that of perylene. Therefore, anhydrides and dianhydrides—the final products of the maleic anhydride cycloaddition to PAH bay regions (to one or both, with the latter observed only for bisanthene)—are intermediates used for the synthesis of diesters and *N*-substituted imides, which are highly or often very highly soluble in organic solvents. Consequently, they could be applied as organic electronics materials, generally as luminophores and photoactive materials. Importantly, it is possible to transform the anhydride motif into the *N*-substituted imide motif through the cycloaddition of different dienophiles, e.g., maleic anhydride or *N*-substituted maleimides, to the other side of the PAH molecule, which leads to coronene derivatives. An important limitation to the combination of two reactions, namely, the cycloaddition of maleic anhydride to PAHs with the subsequent imidization, is the low yields of cycloaddition to alkyl and dialkyl perylene derivatives. Undoubtedly, the cycloaddition of maleic anhydride with further imidization and subsequent cycloaddition reactions “on the other side” is a key synthetic tool when it comes to π-expansion and other modifications of the perylene scaffold. The cycloaddition reactions of functionalized benzynes and other arynes to perylene and its derivatives are also very promising because the products of such reactions can be subjected to further modifications. The spectacular thermally or photochemically initiated domino and cascade HDDA cyclotrimerization–cycloaddition reactions, in which arynes are generated thermally or photochemically from diynes and polyynes via cyclotrimerization, should arouse special interest. Importantly, not only the bare perylene core but also perylene derivatives (e.g., peryleneteracarboxylic acid tetraesters, which can be easily transformed into diimides) undergoes the cycloaddition of arynes. The recently developed cycloaddition of diaryl acetylenes to perylene and PBI bay regions, leading to diarylbenzo[*ghi*]perylenes and diaryl-benzo-PBI, deserve special attention. In fact, products of this reaction can be transformed via cycloaddition “from the other side” of perylene using, e.g., benzyne, benzo-diyne, and maleic anhydride, and via the Scholl reaction. The latter method seems to be especially interesting in the context of the functionalized small pieces of graphene obtained. The reaction of perylene with a benzo-diyne precursor, i.e., 1,2-bis(trimethylsilyl)-5,6-trifluorosulphonato benzene, is also extremely interesting and promising; the final product of this cycloaddition is still the aryne precursor, which can be subjected to tandem aryne generation–*Pd*-catalyzed cyclotrimerization, which results in a triphenylene derivative with perylene motifs (which can be considered small pieces of graphene). The potential for this reaction is the application of functionalized derivatives of benzoperylene, e.g., the ones obtained via the cycloaddition of diarylacetylenes to the bay regions of perylene and perylene bisimides. Moreover, aryne-diyne (e.g., benzo-diyne) cycloaddition can be used to functionalize the graphene surface: after binding to the surface with one triple bond from the aryne-diyne moiety, the second triple bond can be used for further graphene surface functionalization. The recently discovered domino-type reaction between perylene and 1,4-diaryl-13-butadiynes, leading to π-expanded PAHs, creates extraordinary and quite novel opportunities. The products of such reactions can be used for the synthesis of more complex nanographenes via the Scholl reaction, the cycloaddition of arynes, or, in particular, cycloaddition–cyclotrimerization. In the third case, in the first stage of monocycloaddition to the second bay position of perylene derivatives, a precursor of diaryne, e.g., benzo-diyne, is attached. The product of this reaction, namely, a coronene derivative, is still an aryne precursor, and it can be used for the synthesis of nanographene via aryne generation–aryne dimerization or cyclotrimerization. In turn, the combination of the cycloaddition of azenedicarboxylates to perylene, perylene bisimides, and some other PAHs, with the hydrolysis of ester groups to COOH groups, can be a ligand source: derivatives of benzo[*ghi*]perylenedicarboxylic acid. Transition metal complexes, e.g., *Ru*, *Ir*, and *Fe*, with ligands obtained via the cycloaddition of azodicarboxylates, with the subsequent hydrolysis of COOR groups into COOH groups, should exhibit interesting optical properties because of the presence of an extended π-electron system. Since the cycloaddition of diazenedicarboxylates can take place in both bay regions of perylene, it could be applied as an attractive method for the synthesis of 1,2-diazobenzo[*ghi*]perylene and 1,2,6,7-tetrazocoronene derivatives. The cycloaddition reaction of acetylenedicarboxylates is of similar importance to the cycloaddition of azodicarboxylates because of the spectacular improvement of this reaction, i.e., quantitative perylene conversion. The cycloaddition of acetylene (gaseous or generated from precursors, for instance, from nitroethene) can be applied for growing carbon nanotubes; at least, this was the intention of the authors of publications devoted to this reaction. Unfortunately, the development of this concept has not been reported; it seems to be unattractive, perhaps because of the risk of acetylene explosion. In our opinion, cycloadditions to the bay regions of bisanthene (phenanthro[1,10,9,8-*opqra*]perylene) and its derivatives, e.g., substituted in the 7- and 14-positions with t-butyl or mesityl groups, have a special application potential, especially when it comes to the synthesis of small pieces of graphene. As shown by the calculations, the activation energy of the cycloaddition of acetylene is much lower for bisanthene than for perylene. Therefore, the DA cycloaddition of various dienophiles to bisanthene, including acetylene, occurs easily, often in both bay regions, with high or even quantitative conversion. Due to the possibility of reducing the quinone-system to other aromatic systems and introducing solubilizing groups, the cycloaddition of quinones is also very interesting. Above all, the most worthwhile possibility is the combination of large structures to obtain small graphene pieces. The transformation of quinone moieties into diimines, as is known for *p*-benzoquinone, should attract special interest because it provides an ability to create molecular and polymer nanomaterials with interesting photophysical properties [209]. The reactions of primary amines with products of *p*-benzoquinone cycloaddition to perylene and other PAHs are not known, so far. In conclusion, the APEX strategy, represented by the DA cycloaddition of various dienophiles to the bay regions of perylene and other PAHs, has a constantly growing application potential when it comes to modifications of polyaromatic hydrocarbons and the properties of their functionalized derivatives, especially the combination of this reaction with other ones, e.g., imidization of the anhydride motif, cycloaddition to the bay region on the other side of the PAH molecule, the Scholl reaction, and others.

## 14. Conclusions

In this review, all Diels–Alder cycloaddition reactions of various dienophiles to the bay regions of perylene and its derivatives, e.g., benzoperylene, perylene bisimide, perylene tetracarboxylic acid tetraesters, bisanthene, and others, were discussed. This type of one-step annulative π-extension (APEX) strategy has emerged as a powerful and efficient synthetic method for the construction of polycyclic aromatic hydrocarbons and nanographenes, including functionalized ones, and π-extended fused heteroarenes. Importantly, this cycloaddition reaction is always combined with cycloadduct aromatization via hydrogen evolution, oxidative dehydrogenation, transfer of dihydrogen from cycloadducts to the second molecule of the dienophile, and elimination of XY molecules (for dienophiles of the XCH=CHY type). Cycloadditions of ethylenic dienophiles (maleic anhydride, *N*-substituted maleimides, vinyl sulfoxide, alkyl acrylates, and fumarodinitrile, quinones), -N=N-, i.e., diazo-dienophiles (azodicarboxylates and triazolinediones) and acetylenic dienophiles (acetylene, acetylenedicarboxylates, diarylacetylenes, and arynes), were described. This subject was discussed from the organic synthesis point of view but supported by theoretical calculations. The in silico chemistry aspect of these reactions has been studied since 2009. Due to the results of DFT studies, i.e., calculations of activation energies, it was possible to explain the very diverse reactivities of some PAHs to different dienophiles. Calculated activation energy values for the cycloaddition of various acetylenic dienophiles also allows rational synthesis planning, particularly the reaction temperature. For example, the activation energies for the reactions of benzyne, acetylenedicarboxylate, and diphenylacetylene with perylene are 6, 35, and 42 kcal/mol, respectively. These values elucidate why cycloaddition takes place effectively for these dienophiles at different temperatures, i.e., 60 °C, 180 °C, and 280 °C, respectively. Whether the cycloaddition occurs in one or both bay regions of a PAH molecule depends on the reactivity of the diene and dienophile. Perylene is, for example, much more reactive than benzoperylene (which has been examined for the cycloaddition of maleimide by means of kinetic studies) but much less reactive than bisanthene. Therefore, in the case of the latter diene, the cycloaddition process almost always occurs on both sides, and when using acetylene or maleic anhydride, for example, it is quantitative. For perylene and its derivatives (PBI, dialkyl and dicyanoperylenes, and perylene tetracarboxylic acid tetraesters), cycloaddition takes place in one bay region, except for particularly reactive dienophiles (maleimides, triazolinediones, and azodicarboxylates). When it comes to dienophiles, the most reactive are azodicarboxylates, *N*-substituted triazolinediones, and *N*-substituted maleimides: applying them to the cycloaddition of perylene or its derivatives (PBI and dialkylperylene) results in diadduct formation. Importantly, after the cycloaddition of maleimides to perylene, the cycloaddition of maleic anhydride on the other side of perylene is possible, which is particularly important for the synthesis of coronene derivatives. Benzyne is also a very reactive dienophile (i.e., the activation energy of the cycloaddition reaction of benzyne to perylene is very low), but the final product of this reaction, namely, naphthoperylene, is not very reactive, and therefore, the cycloaddition to only one bay region of perylene or its derivatives was observed. Only for bisanthene does the cycloaddition of arynes take place on both sides, but the monoaddition product is predominant. The [4 + 2] cycloaddition reaction combined with cycloadduct aromatization is an atom economical strategy that allows the significant modification of the base structure and the production of the expected properties of the π-extended target structure. It is possible to modify and even tune the properties (e.g., emission and conductive, self-assembly) of target molecules while maintaining the unusual features, particularly the thermal and chemical durability, of perylene and many of its derivatives. Therefore, there is no doubt that the APEX strategy, represented by cycloaddition to PAHs’ bay regions, is an important, attractive, and promising tool for modifying (π-expanding) both unfunctionalized and functionalized PAHs. When it comes to the importance and potential applications of various types of cycloaddition reactions, the cycloaddition of maleic anhydride, as a prelude to further transformations, including cycloaddition on the other side of the molecule (after imidization), is, and will be, very essential. In addition, the cycloaddition of arynes and biarynes (especially those generated from polyynes) and the recently described cycloaddition of diarylacetylenes and 1,4-diaryl-1,3-diarylabutadiynes to perylene and PBI bay regions are very important and promising. The latter is a domino-type cycloaddition–cycloisomerization reaction, in which in the first-stage Diels–Alder cycloaddition of the diyne to the perylene bay region takes place analogously to disubstituted acetylene (only one of the triple bonds undergoes cycloaddition). In the second stage, the resulting masked dienyne undergoes a rapid cycloaromatization reaction, resulting in a π-extended PAH. Reactions that involve arynes, aryne-diynes, diarylacetylenes, and diarylbutadiynes can be used for the synthesis of pieces of functionalized graphene (nanographenes), e.g., via an additional DA cycloaddition on the other side of the PAH molecule, dimerization, or cyclotrimerization.

## Figures and Tables

**Table 1:** Relative reactivity of perylene and their benzo-, naphtho-, and dibenzoderivatives in the DA cycloaddition of phthalic anhydride to the bay region [201].

PAH Structure	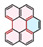	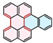	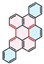	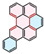	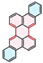	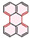
Relative reaction rate	1	7	9	38	175	220

## References

[1] Sun Z., Ye Q., Chi C., Wu J. (2012). Low band gap polycyclic hydrocarbons: From closed-shell near infrared dyes andsemiconductors to open-shell radicals. Chem. Soc. Rev..

[2] Li C., Liu M., Pschirer N.G., Baumgarten M., Müllen K. (2010). Polyphenylene-Based materials for organic photovoltaics. Chem. Rev..

[3] Fermi A., Orfanos I., Avramopoulos A., De Leo F., Demitri N., Bergamini G., Ceroni P., Papadopoulos M.G., Couris S., Bonifazi D. (2017). Tailoring colors by O-annulation of polycyclic aromatic hydrocarbons. Chem. Eur. J..

[4] Wu J., Pisula W., Müllen K. (2007). Graphenes as potential material for electronics. Chem. Rev..

[5] Bendikov M., Wudl F. (2004). Tetrathiafulvalenes, oligoacenenes, and their buckminsterfullerene derivatives:  The brick and mortar of organic electronics. Chem. Rev..

[6] Houk K.N., Lee P.S., Nendel M. (2001). Polyacene and cyclacene geometries and electronic structures: Bond equalization, vanishing band gaps, and triplet ground states contrast with polyacetylene. J. Org. Chem..

[7] Purushothaman B., Bruzek M., Parkin S.R., Miller A.-F., Anthony J.E. (2011). Synthesis and structural characterization of crystalline nonacenes. Angew. Chem. Int. Ed..

[8] Dong D., Fang D., Li H., Zhu C., Zhao X., Li J., Jin L., Xie L., Chen L., Zhao J. (2017). C−H direct arylated 6h -indolo[2,3-b]quinoxaline derivative as a thickness-dependenthole-injection layer. Chem. Asian J..

[9] Zhang Q., Xiao J., Yin Z., Duong H.M., Qiao F., Boey F., Hu X., Zhang H., Wudl F. (2011). Synthesis, characterization, and physical properties of a conjugated heteroacene: 2-methyl-1,4,6,7,8,9-hexaphenylbenz(g)isoquinolin-3(2H)-one (BIQ). Chem. Asian J..

[10] Li G., Wu Y., Gao J., Wang C., Li J., Zhang H., Zhao Y., Zhao Y., Zhang Q. (2012). Synthesis and physical properties of four hexazapentacene derivatives. J. Am. Chem. Soc..

[11] Zhao J., Li R., Ai W., Dong D., Li J., Chen L., Xie L., Yu T., Huang W. (2016). Pi-Extended diindole-fused azapentacenone: Synthesis, characterization, and photophysical and lithium-storage properties. Chem. Asian J..

[12] Xiao J., Duong H.M., Liu Y., Shi W., Ji L., Li G., Li S., Liu X.-W., Ma J., Wudl F. (2012). Synthesis and structure characterization of a stable nonatwistacene. Angew. Chem. Int. Ed..

[13] Gu P.Y., Zhou F., Gao J., Li G., Wang C., Xu Q.-F., Zhang Q., Lu J.-M. (2013). Synthesis, characterization, and nonvolatile ternary memory behavior of a larger heteroacene with nine linearly fused rings and two different heteroatoms. J. Am. Chem. Soc..

[14] Bunz U.H.F. (2015). The larger linear N-heteroacenes. Acc. Chem. Res..

[15] Weil T., Vosch T., Hofkens J., Peneva K., Müllen K. (2010). The rylene colorant family—Tailored nanoemitters for photonics research and applications. Angew. Chem. Int. Ed..

[16] Huang C., Barlow S., Marder S.R. (2011). Perylene-3,4,9,10-tetracarboxylic acid diimides: Synthesis, physical properties, and use in organic electronics. J. Org. Chem..

[17] Li C., Wonneberger H. (2012). Perylene imides for organic photovoltaics: Yesterday, today, and tomorrow. Adv. Mater..

[18] Kozma E., Catellani M. (2013). Perylene diimides based materials for organic solar cells. Dyes Pigments.

[19] Lin Y., Zhan X. (2016). Oligomer molecules for efficient organic photovoltaics. Acc. Chem. Res..

[20] Zhan X., Facchetti A., Barlow S., Marks T.J., Ratner M.A., Wasielewski M.R., Marder S.R. (2011). Rylene and related diimides for organic electronics. Adv. Mater..

[21] Debije M.G., Chen Z., Piris J., Neder R.B., Watson M.M., Müllen K., Würthner F. (2005). Dramatic increase in charge carrier lifetime in a liquid crystalline perylene bisimide derivative upon bay substitution with chlorine. J. Mater. Chem..

[22] Herbst W., Hunger K. (2006). Perylene and perinone pigments. Industrial Organic Pigments: Production, Properties, Applications.

[23] Markiewicz J.T., Wudl F. (2015). Perylene, oligorylenes, and aza-analogs. ACS Appl. Mater. Interfaces.

[24] Lee S.K., Zu Y., Herrmann A., Geerts Y., Müllen K., Bard A.J. (1999). Electrochemistry, spectroscopy and electrogenerated chemiluminescence of perylene, terrylene, and quaterrylene diimides in aprotic solution. J. Am. Chem. Soc..

[25] Quante H., Müllen K. (1995). Quaterrylenebis (dicarboximides). Angew. Chem. Int. Ed. Engl..

[26] Cotlet M., Vosch T., Habuchi S., Weil T., Müllen K., Hofkens J., De Schryver F. (2005). Probing intramolecular förster resonance energy transfer in a naphthaleneimide–peryleneimide–terrylenediimide-based dendrimer by ensemble and single-molecule fluorescence spectroscopy. J. Am. Chem. Soc..

[27] Lewandowska U., Zajączkowski W., Chen L., Bouillière F., Wang D., Koynov K., Pisula W., Müllen K., Wennemers H. (2014). Hierarchical supramolecular assembly of sterically demanding π-systems byconjugation with oligoprolines. Angew. Chem. Int. Ed..

[28] Rocard L., Berezin A., De Leo F., Bonifazi D. (2015). Templated chromophore assembly by dynamic covalent bonds. Angew. Chem. Int. Ed..

[29] Zhylitskaya H., Cybińska J., Chmielewski P., Lis T., Stępień M. (2016). Bandgap engineering in π-extended pyrroles. A modular approach to electron-deficient chromophores with multi-redox activity. J. Am. Chem. Soc..

[30] Maggini L., Bonifazi D. (2012). Hierarchised luminescent organic architectures: Design, synthesis, self-assembly, selforganisation and functions. Chem. Soc. Rev..

[31] Ajayaghosh A., Praveen V.K., Vijayakumar C. (2008). Organogels as scaffolds for excitation energy transfer and light harvesting. Chem. Soc. Rev..

[32] Babu S.S., Aimi J., Ozawa H., Shirahata N., Saeki A., Seki S., Ajayaghosh A., Möhwald H., Nakanishi T. (2012). Solvent-Free luminescent organic liquids. Angew. Chem. Int. Ed..

[33] Bhosale R., Míšek J., Sakai N., Matile S. (2010). Supramolecular n/p-heterojunction photosystems with oriented multicolored antiparallel redox gradients (OMARG-SHJs). Chem. Soc. Rev..

[34] Müllen K., Rabe J.P. (2008). Nanographenes as active components of single-molecule electronics and how ascanning tunneling microscope puts them to work. Acc. Chem. Res..

[35] Dai L. (2013). Functionalization of graphene for efficient energy conversion and storage. Acc. Chem. Res..

[36] Stępień M., Gońka E., Żyła M., Sprutta N. (2017). Heterocyclic nanographenes and other polycyclic heteroaromatic compounds: Synthetic routes, properties, and applications. Chem. Rev..

[37] Ito H., Ozaki K., Itami K. (2017). Annulative π-extension (APEX): Rapid access to fused arenes, heteroarenes, and nanographenes. Angew. Chem. Int. Ed..

[38] Hasobe T. (2012). Photo- and electro-functional self-assembled architectures of porphyrins. Phys. Chem. Chem. Phys..

[39] Hasobe T. (2010). Supramolecular nanoarchitectures for light energy conversion. Phys. Chem. Chem. Phys..

[40] Nakano M., Mori H., Shinamura S., Takimiya K. (2012). Naphtho[2,3-b:6,7-b′]dichalcogenophenes: Syntheses, characterizations, and chalcogeneatom effects on organic field-effect transistor and organic photovoltaic devices. Chem. Mater..

[41] Wang X., Zhi L., Tsao N., Tomović Ż., Li J., Müllen K. (2008). Transparent carbon films as electrodes in organic solar cells. Angew. Chem. Int. Ed..

[42] Zhang G., Zhao J., Chow P.C.Y., Jiang K., Zhang J., Zhu Z., Zhang J., Huang F., Yan H. (2018). Nonfullerene acceptor molecules for bulk heterojunction organic solar cells. Chem. Rev..

[43] Sawabe K., Imakawa M., Nakano M., Yamao T., Hotta S., Iwasa Y., Takenobu T. (2012). Current-Confinement structure and extremely high current density in organic light-emitting transistors. Adv. Mater..

[44] Zhao B., Liu B., Png R.Q., Zhang K., Lim K.A., Luo J., Shao J., Ho P.K.H., Chi C., Wu J. (2010). New discotic mesogens based on triphenylene-fused triazatruxenes: Synthesis, physical properties, and self-assembly. Chem. Mater..

[45] Más-Montoya M., Ortiz R.P., Curiel D., Espinosa A., Allain M., Facchetti A., Marks T.J. (2013). Isomeric carbazolocarbazoles: Synthesis, characterization and comparative study inorganic field effect transistors. J. Mater. Chem. C.

[46] Dadvand A., Moiseev A.G., Sawabe K., Sun W.-H., Djukic B., Chung I., Takenobu T. (2012). Maximizing field-effect mobility and solid-state luminescence in organic semiconductors. Angew. Chem. Int. Ed..

[47] Verma S., Manna M.K., Pandey S.K., Das A.K., Mukherjee S. (2014). Benzo[ghi]perylene monoimide based photosensitive lamellar Cd-doped ZnO nanohybrids. RSC Adv..

[48] Manna M.K., Aaryashree Verma S., Mukherjee S., Das A.K. (2016). Lamellar peptide–cadmium-doped zinc oxide nanohybrids that emit white ligh. ChemPlusChem.

[49] Zollinger H. (2001). Colorants derived from perylene and other polycyclic aromatic compounds. Color Chemistry.

[50] Zhang B., Soleimaninejad H., Jones D.J., White J.M., Ghiggino K.P., Smith T.A., Wong W.W.H. (2017). Highly fluorescent molecularly insulated perylene diimides: Effect of concentration on photophysical properties. Chem. Mater..

[51] Meng D., Sun D., Zhong C., Liu T., Fan B., Huo L., Li Y., Jiang W., Choi H., Kim T. (2016). High-Performance solution-processed non-fullerene organic solar cells based on selenophene-containing perylene bisimide acceptor. J. Am. Chem. Soc..

[52] Li G., Zhao Y., Li J., Cao J., Zhu J., Sun X.W., Zhang Q. (2015). Synthesis, characterization, physical properties, and OLED application of single BN fused perylene diimide. J. Org. Chem..

[53] Endres A.H., Schaffroth M., Paulus F., Reiss H., Wadepohl H., Rominger F., Kramer R., Bunz U.H.F. (2016). Coronene-Containing N-heteroarenes: 13 rings in a row. J. Am. Chem. Soc..

[54] Kong X., Gao J., Ma T., Wang M., Zhang A., Shi Z., Wei Y. (2012). Facile synthesis and replacement reactions of mono-substituted perylene bisimide dyes. Dyes Pigments.

[55] Wang R., Shi Z., Zhang C., Zhang A., Chen J., Guo W., Sun Z. (2013). Facile synthesis and controllable bromination of asymmetrical intermediates of perylenemonoanhydride/monoimide diester. Dyes Pigments.

[56] Ma Y., Shi Z., Zhang A., Li J., Wei X., Jiang T., Li Y., Wang X. (2016). Self-Assembly, optical and electrical properties of five membered O- or S-heterocyclic annulated perylene diimides. Dyes Pigments.

[57] Fan Q., Cheng K., Yang Z., Zhang R., Yang M., Hu X., Ma X., Bu L., Lu X., Xiong X. (2015). Perylene-diimide-based nanoparticles as highly efficient photoacoustic agents for deep brain tumor imaging in living mice. Adv. Mater..

[58] Chen W., Yang X., Long G., Wan X., Chen Y., Zhang Q. (2015). A perylene diimide (PDI)-based small molecule with tetrahedral configuration as a nonfullerene acceptor for organic solar cells. J. Mater. Chem. C.

[59] Würther F., Saha-Mӧller C.R., Fimmel B., Leowanawat P., Schmidt D. (2016). Perylene bisimide dye assemblies as archetype functional supramolecular materials. Chem. Rev..

[60] Mei J., Leung N.L., Kwok R.T., Lam J.W., Tang B.Z. (2015). Aggregation-Induced emission: Together we shine, united we soar. Chem. Rev..

[61] Helin W., Lingcheng C., Zhenbo Z., Yi X. (2017). Aryl-Bisalkynyl bridged perylene diimides dimers: Efficient synthesis, properties andimproved electron mobilities. Dyes Pigments.

[62] Sun J., Wang M., Xu P., Zhang S., Shi Z. (2012). Synthesis of water-soluble perylene dicarboximide derivatives containing pyridineoxide groups. Synth. Commun..

[63] Zhang C., Liu T., Zeng W., Xie D., Luo Z., Sun Y., Yang C. (2017). Thienobenzene-fused perylene bisimide as a non-fullerene acceptor for organic solar cellswith a high open-circuit voltage and power conversion efficiency. Mater. Chem. Front..

[64] Gong Y., Chang K., Chen C., Han M., Zhan X., Min J., Jiao X., Li Q., Li Z. (2019). Pyrene-Fused PDI based ternary solar cells: High power conversion efficiency over 10%, and improved device thermal stability. Mater. Chem. Front..

[65] McAfee S.M., Dayneko S.V., Hendsbee A.D., Josse P., Blanchard P., Cabanetos C., Welch G.C. (2017). Applying direct heteroarylation synthesis to evaluate organic dyes as the core component in PDI-based molecular materials for fullerene-free organic solar cells. J. Mater. Chem. A.

[66] Luo Z., Liu T., Cheng W., Wu K., Xie D., Huo L., Sun Y., Yang C. (2018). A three-dimensional thiophene-annulated perylene bisimide as a fullerene-free acceptor fora high performance polymer solar cell with the highest PCE of 8.28% and a VOC over 1.0 V. J. Mater. Chem. C.

[67] Zhao Y., Wang H., Zeng W., Xia S., Zhou F., Chen H., He F., Yang C. (2018). Regulating the optoelectronic properties of small molecule donors with multiple alternative electron-donor and acceptor units for organic solar cells. J. Mater. Chem. A.

[68] Lin K., Wang S., Wang Z., Yin Q., Liu X., Jia J., Jia X., Luo P., Jiang X., Duan C. (2018). Electron acceptors with a truxene core and perylene diimide branches for organic solar cells: The effect of ring-fusion. Front. Chem..

[69] Ostroverkhova O. (2018). Nitroxide-Mediated radical polymerizations. Handbook of Organic Materials for Electronic and Photonic Devices.

[70] Nowak-Król A., Würthner F. (2019). Progress in the synthesis of perylene bisimide dyes. Org. Chem. Front..

[71] Bressan G., Green D., Chan Y., Bulman Page P.C., Jones G.A., Meech S.R., Heisler I.A. (2019). One- to two-exciton transitions in perylene bisimide dimer revealed by two dimensional electronic spectroscopy. J. Phys. Chem. A.

[72] Avellanal-Zaballa E., Durán-Sampedro G., Prieto-Castañeda A., Agarrabeitia A.R., García-Moreno I., López-Arbeloa I., Bañuelos J., Ortiz M.J. (2017). Rational molecular design enhancing the photonic performance of red-emitting perylene bisimide dyes. Phys. Chem. Chem. Phys..

[73] Guo Z., Zhang X., Wang Y., Li Z. (2019). Supramolecular self-assembly of perylene bisimide derivatives assisted by various groups. Langmuir.

[74] Khan Q.U., Tian G., Bao L., Qi S., Wu D. (2018). Highly uniform supramolecular nano-films derived from carbazole-containing perylene diimide via surface-supported self-assembly and their electrically bistable memory behavior. New J. Chem..

[75] Yamauchi M., Masuo S. (2019). Colloidal quantum dot arrangement assisted by perylene bisimide self-assembly. Chem. Eur. J..

[76] Chen S., Slattum P., Wang C., Zang L. (2015). Self-Assembly of perylene imide molecules into 1D nanostructures: Methods, morphologies, and applications. Chem. Rev..

[77] Oh J.H., Lee H.W., Mannsfeld S., Stoltenberg R.M., Jung E., Jin Y.W., Kim J.M., Yoo J.B., Bao Z. (2009). Solution-Processed, high-performance n-channel organic microwire transistors. Proc. Natl. Acad. Sci. USA.

[78] Würther F. (2004). Perylene bisimide dyes as versatile building blocks for functional supramolecular Architectures. Chem. Commun..

[79] Jones B.A., Ahrens M.J., Yoon M.H., Facchetti A., Marks T.J., Wasilewski M.R. (2004). High-Mobility air-stable n-type semiconductors with processing versatility: Dicyanoperylene-3,4:9,10-bis(dicarboximides). Angew. Chem. Int. Ed..

[80] Wen Y.G., Liu Y.Q. (2010). Recent progress in n-channel organic thin-film transistors. Adv. Mater..

[81] Mende-Schmidt L., Fechtenkötter A., Müllen K., Moons E., Friend R.H., MacKenzie J.D. (2001). Self-Organized discotic liquid crystals for high-efficiency organic photovoltaics. Science.

[82] Lin Y., Wang Y., Wang J., Hou J., Li Y., Zhu D., Zhan X. (2014). A Star-shaped perylene diimide electron acceptor for high-performance organic solar cells. Adv. Mater..

[83] Zhan C., Yao J. (2016). More than conformational “twisting” or “coplanarity”: Molecular strategies for designing high-efficiency nonfullerene organic solar cells. Chem. Mater..

[84] Chen N., Lu J., Wang D., Zheng C., Wu H., Zhang H., Gao D. (2018). A double-cable poly(fluorene-alt-thiophene) with bay-substituted perylenediimide pendants: An efficient interfacial material in bulk-heterojunction solar cells. Macromolecules.

[85] Matussek M., Filapek M., Gancarz P., Krompiec S., Małecki J.G., Kotowicz S., Siwy M., Maćkowski S., Chrobok A., Schab-Balcerzak E. (2018). Synthesis and photophysical properties of new perylene bisimide derivatives for application as emitting materials in OLEDs. Dyes Pigments.

[86] Meng X., Zhu W., Tian H., Sun S.-S., Dalton L.R. (2016). Perylene bisimide derivatives for organic light-emitting diodes. Introduction to Organic Electronic and Optoelectronic Materials and Devices.

[87] Mako T.L., Racicot J.M., Levine M. (2019). Supramolecular luminescent sensors. Chem. Rev..

[88] Shoyama K., Mahl M., Seifert S., Würthner F. (2018). A general synthetic route to polycyclic aromatic dicarboximides by palladiumcatalyzed annulation reaction. J. Org. Chem..

[89] Anthony J.E., Facchetti A., Heeney M., Marder S.R., Zhan X. (2010). n-Type organic semiconductors in organic electronics. Adv. Mater..

[90] Jung B.J., Tremblay N.J., Yeh M.L., Katz H.E. (2011). Molecular design and synthetic approaches to electron-transporting organic transistorsemiconductors. Chem. Mater..

[91] Sonawane S.L., Asha S.K. (2013). Fluorescent cross-linked polystyrene perylenebisimide/oligo(p-phenylenevinylene)microbeads with controlled particle size, tunable colors, and high solid state emission. ACS Appl. Mater. Interfaces.

[92] Sun J.P., Hendsbee A.D., Dobson A.J., Welch G.C., Hill I.G. (2016). Perylene diimide based all small-molecule organic solar cells: Impact of branched-alkylside chains on solubility, photophysics, self-assembly, and photovoltaic parameters. Org. Electron..

[93] McAfee S.M., Topple J.M., Hill I.G., Welch G.C. (2015). Key components to the recent performance increases of solution processed non-fullerenesmall molecule acceptors. J. Mater. Chem. A.

[94] Nielsen C.B., Holliday S., Chen H.Y., Cryer S.J., McCulloch I. (2015). Non-Fullerene electron acceptors for use in organic solar cells. Acc. Chem. Res..

[95] Lin Y., Wang J., Zhang Z.-G., Bai H., Li Y., Zhu D., Zhan X. (2015). An electronacceptor challenging fullerenes for efficient polymer solar cells. Adv. Mater..

[96] Lin Y., Zhao F., He Q., Huo L., Wu Y., Parker T.C., Ma W., Sun Y., Wang C., Zhu D. (2016). High-Performance electron acceptor with thienyl side chains for organic photovoltaics. J. Am. Chem. Soc..

[97] Lin Y., He Q., Zhao F., Huo L., Mai J., Lu X., Su C.-J., Li T., Wang J., Zhu J. (2016). A facile planar fused-ring electron acceptor for as-cast polymer solar cells with 8.71% efficiency. J. Am. Chem. Soc..

[98] Schmidt C.D., Lang N., Jux N., Hirsch A. (2011). A facile route to water-soluble coronenes and benzo[ghi]perylenes. Chem. Eur. J..

[99] Schulze M., Philipp M., Waigel W., Schmidt D., Würthner F. (2016). Library of azabenz-annulated core-extended perylene derivatives with diverse substitution patterns and tunable electronic and optical properties. J. Org. Chem..

[100] Vollbrecht J., Wiebeler C., Neuba A., Bock H., Schumacher S., Kitzerow H. (2016). Bay-Extended distorted perylene esters showing visible luminescence after ultraviolet excitation: Photophysical and electrochemical analysis. J. Phys. Chem. C.

[101] Wang R., Li G., Zhang A., Wang W., Cui G., Zhao J., Shi Z., Tang B. (2017). Efficient energy-level modification of novel pyran-annulated perylene diimides for photocatalytic water splitting. Chem. Commun..

[102] Gupta R.K., Dey A., Singh A., Iyer P.K., Sudhakar A.A. (2019). Heteroatom bay-annulated perylene bisimides: New materials for organic field effect transistors. ACS Appl. Electron. Mater..

[103] Zang Y., Li C.Z., Chueh C.C., Williams S.T., Jiang W., Wang Z.-H., Yu J.S., Jen A.K.Y. (2014). Integrated molecular, interfacial, and device engineering towards high-performance non-fullerene based organic solar cells. Adv. Mater..

[104] Liu T., Ge Y., Sun B., Fowler B., Li H., Nuckolls C., Xiao S. (2019). Synthesis, regioselective bromination, and functionalization of coronene tetracarboxydiimide. J. Org. Chem..

[105] Hendsbee A.D., Sun J.P., Law W.K., Yan H., Hill I.G., Spasyuk D.M., Welch G.C. (2016). Synthesis, self-assembly, and solar cell performance of N-annulated perylene non-fullerene acceptors. Chem. Mater..

[106] Ball M.L., Zhang B., Xu Q., Paley D.W., Ritter V.C., Ng F., Steigerwald M.L., Nuckolls C. (2018). Influence of molecular conformation on electron transport in giant, conjugated macrocycles. J. Am. Chem. Soc..

[107] Zhang J., Li Y., Huang J., Hu H., Zhang G., Ma T., Chow P.C.Y., Ade H., Pan D., Yan H. (2017). Ring-Fusion of perylenediimide acceptor enabling efficient nonfullerene organic solar cells with a small voltage loss. J. Am. Chem. Soc..

[108] Sekida S., Kameyama T., Koga T., Hadano S., Watanabe S., Niko Y. (2018). Highly lipophilic and solid emissive N-annulated perylene bisimide synthesis for facile preparation of bright and far-red excimer fluorescent nano-emulsions with large Stokes shift. J. Photochem. Photobiol. A.

[109] Zhan C., Jiang Y.-Y., Yang M.-Y., Lu L.-H., Xiao S.Q. (2014). Synthesis and optoelectronic properties of a novel molecular semiconductor of dithieno[5,6-b:11,12-b’]coronene-2,3,8,9-tetracarboxylic tetraester. Chin. Chem. Lett..

[110] Chen Z., Li J., Li M., Chen C., Xu S., Tang X., Chen L., Chen R., Huang W. (2018). Synthesis and application of perylene-embedded benzoazoles for small-molecule organic solar cells. Org. Lett..

[111] Gupta R.K., Ulla H., Satyanarayan M.N., Sudhakar A.A. (2018). A perylene-tiazine-based star-shaped green light emitter for organic light emitting diodes. Eur. J. Org. Chem..

[112] Yoshida M., Sakai H., Ohkubo K., Fukuzumi S., Hasobe T. (2017). Inter- and intramolecular electron-transfer reduction properties of coronenediimides derivatives via photoinduced processes. J. Phys. Chem. C.

[113] Gupta R.K., Pathak S.K., Pradhan B., Gupta M., Pal S.K., Sudhakar A.A. (2016). Bay-Annulated perylene tetraesters: A new class of discotic liquid crystals. ChemPhysChem.

[114] Liu X., Chen M., Xiao C., Xue N., Zhang L. (2018). Soluble twisted diarenoperylenes: Synthesis, characterization, and device performance. Org. Lett..

[115] Li X., Wang H., Schneider J.A., Wei Z., Lai W.-Y., Huang W., Wudl F., Zheng Y. (2017). Catalyst-Free one-step synthesis of ortho-tetraaryl perylene diimides for efficient OPV non-fullerene acceptors. J. Mater. Chem. C.

[116] Welsh T.A., Laventure A., Alahmadi A.F., Zhang G., Baumgartner T., Zou Y., Jäkle F., Welch G.C. (2019). Borane incorporation in a non-fullerene acceptor to tune steric and electronic properties and improve organic solar cell performance. ACS Appl. Energy Mater..

[117] Li L., Hong Y.-J., Chen D.-Y., Lin M.J. (2017). A laterally extended perylene hexacarboxylate via Diels-Alder reaction for high-performance organic lithium-ion batteries. Electrochim. Acta.

[118] Schulze M., Steffen A., Würthner F. (2015). Near-IR phosphorescent ruthenium(II) and iridium(III) perylene bisimide metal complexes. Angew. Chem. Int. Ed..

[119] Liu M., Yang J., Lang C., Zhang Y., Zhou E., Liu Z., Guo F., Zhao L. (2017). Fused perylene diimide-based polymeric acceptors for efficient all-polymer solar cells. Macromolecules.

[120] Zhao D., Wu Q., Cai Z., Zheng T., Chen W., Lu J., Yu L. (2016). Electron acceptors based on α-substituted perylene diimide (PDI) for organic solar cells. Chem. Mater..

[121] Takahashi M., Asaba K., Lua T.T., Inuzuka T., Uemura N., Sakamoto M., Sengoku T., Yoda H. (2018). Controllable monobromination of perylene ring system: Synthesis of bay-functionalized perylene dyes. J. Org. Chem..

[122] Kaufmann C., Bialas D., Stolte M., Würther F. (2018). Discrete π-stacks of perylene bisimide dyes within folda-dimers: Insight into long- and short-range exciton coupling. J. Am. Chem. Soc..

[123] Regar R., Mishra R., Mondal P.K., Sankar J. (2018). Metal-Free annulation at the ortho- and bay-positions of perylene bisimide leading to lateral π-extension with strong NIR absorption. J. Org. Chem..

[124] Mao W., Zhang J., Li X., Li C., Tian H. (2017). Regioisomerically pure multiaryl coronene derivatives: Highly efficient synthesis via bay-extended perylene tetrabutylester. Chem. Commun..

[125] Meng D., Liu G., Xiao C., Shi Y., Zhang L., Jiang L., Baldridge K.K., Li Y., Siegel J.S., Wang Z. (2019). Corannurylene pentapetalae. J. Am. Chem. Soc..

[126] Rao K.V., George S.J. (2010). Synthesis and controllable self-assembly of a novel coronene bisimide amphiphile. Org. Lett..

[127] Kelber J., Achard M.F., Durola F., Bock H. (2012). Distorted arene core allows room-temperature columnar liquid-crystal glass with minimal side chains. Angew. Chem. Int. Ed..

[128] Vollbrecht J., Bock H., Wiebeler C., Schumacher S., Kitzerow H. (2014). Polycyclic aromatic hydrocarbons obtained by lateral core extension of mesogenic perylenes: Absorption and optoelectronic properties. Chem. Eur. J..

[129] Schuster N.J., Paley D.W., Jockusch S., Ng F., Steigerwald M.R., Nuckolls C. (2016). Electron delocalization in perylene diimide helicenes. Angew. Chem. Int. Ed..

[130] Eversloh C.L., Li C., Müllen K. (2011). Core-Extended perylene tetracarboxdiimides: The homologous series of coronene tetracarboxdiimides. Org. Lett..

[131] Ito H., Segawa Y., Murakami K., Itami K. (2019). Polycyclic arene synthesis by annulative π-extension. J. Am. Chem. Soc..

[132] Nakamuro T., Kumazawa K., Ito H., Itami K. (2019). Bay-region-selective annulative π-extension (APEX) of perylene diimides with arynes. Synlett.

[133] Zink-Lorre N., Doncel-Giménez A., Font-Sanchis E., Calbo J., Sastre-Santos Á., Ortí E., Fernández-Lázaro F. (2019). Diels-Alder reaction on perylenediimides: Synthesis and theoretical study of core-expanded diimides. Org. Chem. Front..

[134] Dyan O.T., Borodkin G.I., Zaikin P.A. (2019). The Diels-Alder reaction for the synthesis of polycyclic aromatic compounds. Eur. J. Org. Chem..

[135] Clar E. (1932). Über die Konstitution des Perylens; die Synthesen des 2.3,10.11-Dibenz- und des 1.12-Benz-perylens und betrachtungen über die Konstitution des benzanthrons und phenanthrens (Zur kenntnis mehrkerniger aromatischer kohlenwasserstoffe und ihrer abkӧmmlinge, XIV. Mitteil. Ber. Dtsch. Chem. Ges..

[136] Clar E., Zander M.J. (1957). Syntheses of coronene and 1: 2–7: 8-dibenzocoronene. Chem. Soc..

[137] Herrero M.A., Kremsner J.M., Kappe C.O. (2008). Nonthermal microwave effects revisited:  on the importance of internal temperature monitoring and agitation in microwave chemistry. J. Org. Chem..

[138] Manning S.T., Bogen W., Kelly L.A. (2011). Synthesis, characterization, and photophysical study of fluorescent N-substituted benzo[ghi]perylene “swallow tail” monoimides. J. Org. Chem..

[139] Zhou C., Li W., Chen J., Yang M., Li Y., Zhu J., Yu C. (2014). Real-Time fluorometric turn-on assay for protease activity and inhibitor screening with a benzoperylene probe. Analyst.

[140] Yang M., Zhou H., Li Y., Zhang Q., Li J., Zhang C., Zhou C., Yu C. (2017). Peroxidase activity of the coronene bisimide supramolecular architecture and its applications in colorimetric sensing of H_2_O_2_ and glucose. J. Mater. Chem. C.

[141] Alibert-Fouet S., Seguy I., Bobo J.-F., Destruel P., Bock H. (2007). Liquid-Crystalline and electron-deficient coronene oligocarboxylic esters and imides by twofold benzogenic Diels-Alder reactions on perylenes. Chem. Eur. J..

[142] Jain A., Rao K.V., Kulkarni C., George A., George S.J. (2012). Fluorescent coronene monoimide gels via H-bonding induced frustrated dipolar assembly. Chem. Commun..

[143] Hopff H., Schweizer H.R. (1959). Zur kenntnis des coronens. 2. Mitteilung. Dien-anlagerungen in der perylen- und benzperylenreihe. Helv. Chim. Acta.

[144] Hirayama S., Sakai H., Araki Y., Tanaka M., Imakawa M., Wada T., Takenobu T., Hasobe T. (2014). Systematic control of the excited-state dynamics and carrier-transport properties of functionalized benzo[ghi]perylene and coronene derivatives. Chem. Eur. J..

[145] Tokita S., Hiruta K., Kitahara K., Nishi H. (1982). Diels-Alder reaction of dialkyl diazenedicarboxylates with perylenes; a new synthesis of polycyclic aromatic pyridazines. Synthesis.

[146] Ott R., Wiedemann F., Zinke A. (1968). Untersuchungen über perylen und seine derivate, 67. Mitt.: Mehrkernige aromaten durch diensynthesen mit perylen. Mon. Chem..

[147] Fort E.H., Scott L.T. (2010). One-Step conversion of aromatic hydrocarbon bay regions into unsubstituted benzene rings: A reagent for the low-temperature, metal-free growth of single-chirality carbon nanotubes. Angew. Chem. Int. Ed..

[148] Jackson E.P., Sisto T.J., Darzi E.R., Jasti R. (2016). Probing Diels-Alder reactivity on a model CNT sidewall. Tetrahedron.

[149] Fort E.H., Donovan P.M., Scott L.T. (2009). Diels-Alder reactivity of polycyclic aromatic hydrocarbon bay regions: Implications for metal-free growth of single-chirality carbon nanotubes. J. Am. Chem. Soc..

[150] Domingo R.E., Arnó M., Contreras R., Pérez P. (2002). Density Functional Theory study for the cycloaddition of 1,3-butadienes with dimethyl acetylenedicarboxylate. Polar stepwise vs concerted mechanisms. J. Phys. Chem. A.

[151] Krylov I.M., Mailyan A.K., Zotova M.A., Bruneau C., Dixneuf P.H., Osipov S.N. (2014). Access to functionalized α-trifluoromethyl-α-aminophosphonates via intermolecular ene-yne metathesis. Synlett.

[152] Kurpanik A., Matussek M., Szafraniec-Gorol G., Filapek M., Lodowski P., Marcol-Szumilas B., Ignasiak W., Małecki J.G., Machura B., Małecka M. (2020). APEX strategy represented by Diels-Alder cycloadditions—New opportunities for the syntheses of functionalised PAHs. Chem. Eur. J..

[153] Pająk M., Kurpanik A., Zych D., Krompiec S., Matussek M., Marcol B., Filapek M. (2018). Method for Obtaining 1′,2′-Bis(Methoxycarbonyl)-1,12-Benzoperylene or 1′,2′-Bis(Ethoxycarbonyl)-1,12-Benzoperylene. PL Patent.

[154] Krompiec S., Szafraniec-Gorol G., Lodowski P., Matussek M., Marcol-Szumilas B., Ignasiak W. (2018). 1,2-Diarylbenzo[ghi]Perylenes and Method of Preparing Them. PL Patent.

[155] Szafraniec-Gorol G., Matussek M., Krompiec S., Jendrzejewska I., Marcol-Szumilas B., Ignasiak W., Gudwański A. (2019). The Method of Preparation of 1,2-Diarylbenzo[ghi]Perylenes. PL Patent.

[156] Zimmermann G. (2001). Cycloaromatization of open and masked 1,3-hexadien-5-ynes—Mechanistic and synthetic aspects. Eur. J. Org. Chem..

[157] Yang V., Bam R., Catalano V.J., Chalifoux W.A. (2018). Highly regioselective domino benzannulation reaction of buta-1,3-diynes to construct irregular nanographenes. Angew. Chem. Int. Ed..

[158] Chen T.-A., Liu R.-S. (2011). Synthesis of polyaromatic hydrocarbons from bis(biaryl)diynes: Large PAHs with low Clar sextets. Chem. Eur. J..

[159] Liu J., Li B.W., Tan Y.Z., Giannakopoulos A., Sanchez-Sanchez C., Beljonne D., Ruffieux P., Fasel R., Feng X., Müllen K. (2015). Toward cove-edged low band gap graphene nanoribbons. J. Am. Chem. Soc..

[160] Lòpez F., Mascareñas J.L. (2011). Recent developments in gold-catalyzed cycloaddition reactions. Beilstein J. Org. Chem..

[161] Paternò G.M., Chen Q., Wang X.Y., Liu J., Motti S.G., Petrozza A., Feng X., Lanzani G., Müllen K., Narita A. (2017). Synthesis of dibenzo[hi,st]ovalene and its amplified spontaneous emission in a polystyrene matrix. Angew. Chem. Int. Ed..

[162] Hitt D.M., O’Connor J.M. (2011). Acceleration of conjugated dienyne cycloaromatization. Chem. Rev..

[163] Narita A., Wang X.Y., Feng X., Müllen K. (2015). New advances in nanographene chemistry. Chem. Soc. Rev..

[164] Aguilar E., Sanz R., Fernández-Rodríguez M., García-García P. (2016). 1,3-Dien-5-ynes: Versatile building blocks for the synthesis of carbo- and heterocycles. Chem. Rev..

[165] Szafraniec-Gorol G., Krompiec S., Ignasiak W. (2019). Pi-Extended Perylene Derivative and the Method of Its Production. PL Patent.

[166] Szafraniec-Gorol G., Krompiec S., Matussek M., Orszulak L. (2019). Pi-Extended Perylene Derivative and the Method of Its Production. PL Patent.

[167] Stork G., Matsuda K. (1968). Preparation of Benzocoronene and Intermediates. U.S. Patent.

[168] Pavlyuk D.E., Gundala S., Kovalev I.S., Kopchuk D.S., Krinochkin A.P., Budeev A.V., Zyryanov G.V., Venkatapuram P., Rusinov V.L., Chupakhin O.N. (2019). Reactions of perylene with aryne intermediates. Russ. J. Org. Chem..

[169] Fort E.H., Scott L.T. (2011). Gas-phase Diels-Alder cycloaddition of benzyne to an aromatic hydrocarbon bay region: Groundwork for the selective solvent-free growth of armchair carbon nanotubes. Tetrahedron Lett..

[170] Schuler B., Collazos S., Gross L., Meyer G., Pérez D., Guitián E., Peña D. (2014). From perylene to a 22-ring aromatic hydrocarbon in one-pot. Angew. Chem. Int. Ed..

[171] Rüdiger E.C., Porz M., Schaffroth M., Rominger F., Bunz U.H.F. (2014). Synthesis of soluble, alkyne-substituted trideca- and hexadeca-starphenes. Chem. Eur. J..

[172] Pérez D., Peña D., Guitián E. (2013). Aryne Cycloaddition Reactions in the Synthesis of Large Polycyclic Aromatic Compounds. Eur. J. Org. Chem..

[173] Kumarasinghe K.G.U.R., Fronczek F.R., Valle H.U., Sygulla A. (2016). Bis-corannulenoanthracene: An angularly fused pentacene as a precursor for barrelene-tethered receptors for fullerenes. Org. Lett..

[174] Chen L., Zhang C., Wen C., Zhang K., Liu W., Chen Q. (2015). Gold-Catalyzed cyclotrimerization of arynes for the synthesis of triphenylenes. Catal. Commun..

[175] Xu F., Xiao X., Hoye T.R. (2016). Reactions of HDDA-derived benzynes with perylenes: Rapid construction of polycyclic aromatic compounds. Org. Lett..

[176] Xu F., Xiao X., Hoye T.R. (2017). Photochemical hexadehydro-Diels-Alder reaction. J. Am. Chem. Soc..

[177] Xiao X., Hoye T.R. (2018). The domino hexadehydro-Diels-Alder reaction transforms polyynes to benzynes to naphthynes to anthracynes to tetracynes (and beyond?). Nat. Chem..

[178] Kurpanik A., Krompiec S., Marcol-Szumilas B., Łucka J. (2019). The Method of Naphtho-, Anthraceno- and Phenanthro[1,2,3,4-ghi]Perylenes Preparation. PL Patent.

[179] Kurpanik A., Krompiec S., Łucka J. (2019). The Method of Naphtho[1,2,3,4-ghi]Perylene Preparation. PL Patent.

[180] Kurpanik A., Krompiec S., Marcol-Szumilas B., Łucka J. (2019). The Method of Naphtho-, Anthraceno- and Phenanthro[1,2,3,4-ghi]Perylenes Preparation. PL Patent.

[181] Kurpanik A., Krompiec S., Marcol-Szumilas B., Grabowska A. (2019). The Method of Naphtho-, Anthraceno- and Phenanthro[1,2,3,4-ghi]Perylenes Preparation. PL Patent.

[182] Cammidge A.N., Gopee H. (2006). Perylenophthalocyanines. Chem. Eur. J..

[183] Gӧltner C., Pressner D., Müllen K., Spiess H.W. (1993). Liquid-Crystalline perylene derivatives as “Discotic pigments”. Angew. Chem. Int. Ed. Engl..

[184] Schlichting P., Rohr U., Müllen K. (1998). Easy synthesis of liquid crystalline perylene derivatives. J. Mater. Chem..

[185] Jiang W., Qian H., Li Y., Wang Z. (2008). Heteroatom-annulated perylenes: Practical synthesis, photophysical properties, and solid-state packing arrangement. J. Org. Chem..

[186] Kelber J., Achard M.F., Garreau-de Bonneval B., Bock H. (2011). Columnar benzoperylene-hexa- and tetracarboxylic imides and esters: Synthesis, mesophase stabilisation and observation of charge-transfer interactions between electron-donating esters and electron-accepting imides. Chem. Eur. J..

[187] Choi M., Do H.Y. (2014). Synthesis of perylene dianhydride-incorporated main chain polyimides and sequential structural transformation through a dipolar cycloaddition. React. Func. Polym..

[188] Langhals H., Kirner S., Blanke P., Speckbacher M., Langhals H., Kirner S., Blanke P., Speckbacher M. (2002). Core-Extended Perylene Bisimides. U.S. Patent.

[189] Langhals H., Kirner S. (2000). Novel fluorescent dyes by the extension of the core of perylenetetracarboxylic bisimides. Eur. J. Org. Chem..

[190] Krompiec S., Szafraniec-Gorol G., Matussek M., Ignasiak W., Filapek M. (2020). 2,3-Diphenyl-N,N′-bis(2-Ethylhexyl)Benzo[ghi]Perylenediimide and the Method of Its Production. PL Patent.

[191] Krompiec S., Szafraniec-Gorol G., Matussek M., Ignasiak W., Lodowski P. (2020). 2,3-bis[7-(9H-Carbazol-9-yl)-9,9-Dibutylfluoren-2-yl]-N,N′-bis(2-Ethylhexyl)Benzo[ghi]Perylenediimide and the Method of Its Production. PL Patent.

[192] Krompiec S., Szafraniec-Gorol G., Matussek M., Ignasiak W., Lodowski P. (2020). 2,3-bis[N-(2-Ethylhexyl)Phthalimid-4-yl]-N,N′-bis(2-Ethylhexyl)Benzo[ghi]Perylenediimide and the Method of Its Production. PL Patent.

[193] Krompiec S., Szafraniec-Gorol G., Matussek M., Ignasiak W., Filapek M. (2020). 2,3-Diphenyl-N,N′-bis(2,6-Diisopropylphenyl)Benzo[ghi]Perylenediimide and the Method of Its Production. PL Patent.

[194] Kurpanik A., Marcol-Szumilas B., Krompiec S., Gołek B. (2019). Preparation Method of Tetrabenzyl Anthracene[1,2,3,4-ghi]Perylene-7,8,13,14-Tetracarboxylate. PL Patent.

[195] Kurpanik A., Marcol-Szumilas B., Krompiec S., Gołek B. (2019). Preparation Method of Tetrabenzyl Phenanthro[1,2,3,4-ghi]Perylene-6,7,12,13-Tetracarboxylate. PL Patent.

[196] Kurpanik A., Marcol-Szumilas B., Krompiec S., Gołek B. (2019). Preparation Method of Tetrabenzyl Naphtho[1,2,3,4-ghi]Perylene-4,5,10,11-Tetracarboxylate. PL Patent.

[197] Kurpanik A., Marcol-Szumilas B., Krompiec S., Grabowska A., Gołek B. (2019). 1,2-Diphenylbenzo[1,2-j]Coronene and the Method of Its Preparation. PL Patent.

[198] Kurpanik A., Ignasiak W., Krompiec S., Matussek M., Gołek B., Grabowska A. (2019). 1,2-Bis(9,9-Dibutylfluoen-2-yl)Benzo[1,2-j]Coronene and the Method of Its Preparation. PL Patent.

[199] Kurpanik A., Szafraniec-Gorol G., Krompiec S., Matussek M., Gołek B. (2019). 1,2-Bis(N-2-Etylhexylphthalimido-4-yl)Benzo[1,2-j]Coronene and the Method of Its Preparation. PL Patent.

[200] Kurpanik A., Krompiec S. (2020). 1,2-di(Metoxycarbonyl)Benzo[1,2-j]Coronene and Method of Its Preparation. PL Patent.

[201] Zander M. (1969). Relative reaktionsgeschwindigkeiten der maleinsäureanhydrid-addition an kohlenwasserstoffe der perylen- und 1.12-benzperylen-reihe. Liebigs Ann. Chem..

[202] Clar E. (1948). Synthesis of ovalene. Nature.

[203] Jinling L., Baozhan X., Jin P. (2015). Synthesis of bisanthene-based polycyclic aromatic hydrocarbons. Chin. J. Org. Chem..

[204] Fort E.H., Jeffreys M.S., Scott L.T. (2012). Diels-Alder cycloaddition of acetylene gas to a polycyclic aromatic hydrocarbon bay region. Chem. Commun..

[205] Konishi A., Hirao Y., Matsumoto K., Kurata H., Kubo T. (2013). Facile synthesis and lateral π-expansion of bisanthenes. Chem. Lett..

[206] Li J., Jiao C., Huang K.W., Wu J. (2011). Lateral extension of π conjugation along the bay regions of bisanthene through a Diels-Alder cycloaddition reaction. Chem. Eur. J..

[207] Kruse H., Goerigk L., Grimme S. (2012). Why the standard B3LYP/6-31G* model chemistry should not be used in DFT calculations of molecular thermochemistry: Understanding and correcting the problem. J. Org. Chem..

[208] Cao Y., Osuna S., Liang Y., Haddon R.C., Houk K.N. (2013). Diels-Alder reactions of graphene: Computational predictions of products and sites of reaction. J. Am. Chem. Soc..

[209] Lei H., Langlois A., Fortin D., Karsenti P.L., Aly S.M., Harvey P.D. (2017). Renderingcross-Conjugated azophenine derivatives emissive to probe the silent photophysical properties of emeraldine. Phys. Chem. Chem. Phys..

